# Supplementary figures and images for: The nanoCUT&RUN technique visualizes telomeric chromatin in Drosophila
Source: PLoS Genet. 2022 Sep 1;18(9):e1010351. doi: 10.1371/journal.pgen.1010351 (PMC9473618; doi:10.1371/journal.pgen.1010351)

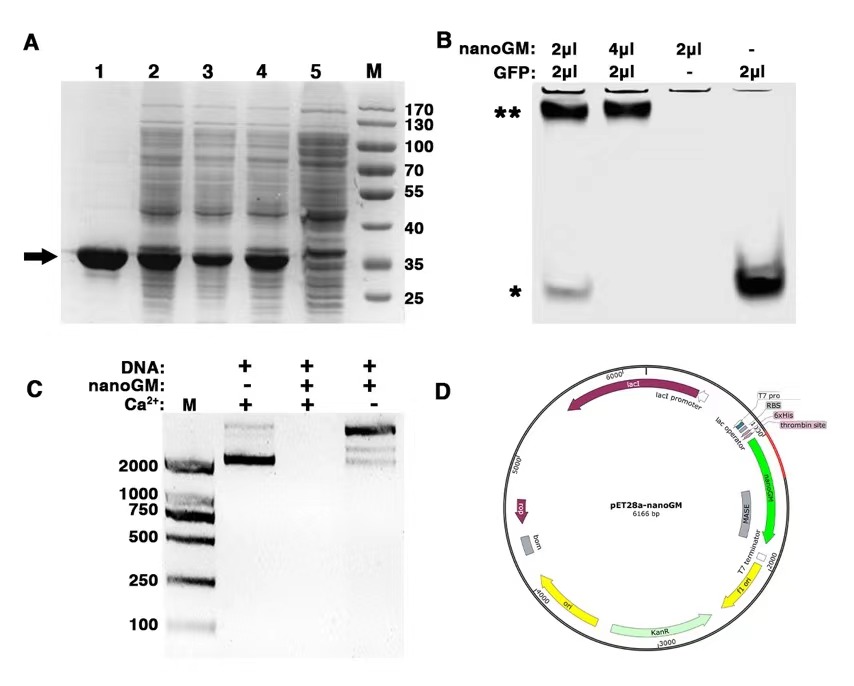

Supplement: S1 Fig — A: purification of the nGFPMNase fusion protein from bacteria. Extracts from different fractions were run on SDS-Page and stained with Coomassie Blue. Lanes 1: purified nGFPMNase; 2: insoluble fraction from bacteria overexpressing nGFPMNase; 3: soluble fraction; 4: total extract from overexpressing bacteria; 5: total extract from uninduced bacterial culture. “M” denotes protein markers with sizes in KD indicated to the right. The arrow marks the running position of nGFPMNase. B: nGFPMNase (nanoGM) binds GFP. GFP fluorescence from a native protein gel is shown with protein components loaded onto each lane shown at the top. Note that nGFPMNase alone does not emit fluorescence. The double star marks the running position of the complex between nGFPMNase and GFP. The single star marks the running position of GFP alone. C: nGFPMNase (nanoGM) digests DNA in the presence of calcium. Plasmid DNA was mixed with purified nGFPMNase in the nuclease digestion buffer with or without calcium. “M” denotes DNA markers with sizes indicated to the right. D: map of the nGFPMNase expression plasmid. (JPEG) [file pgen.1010351.s001.jpeg]

**A**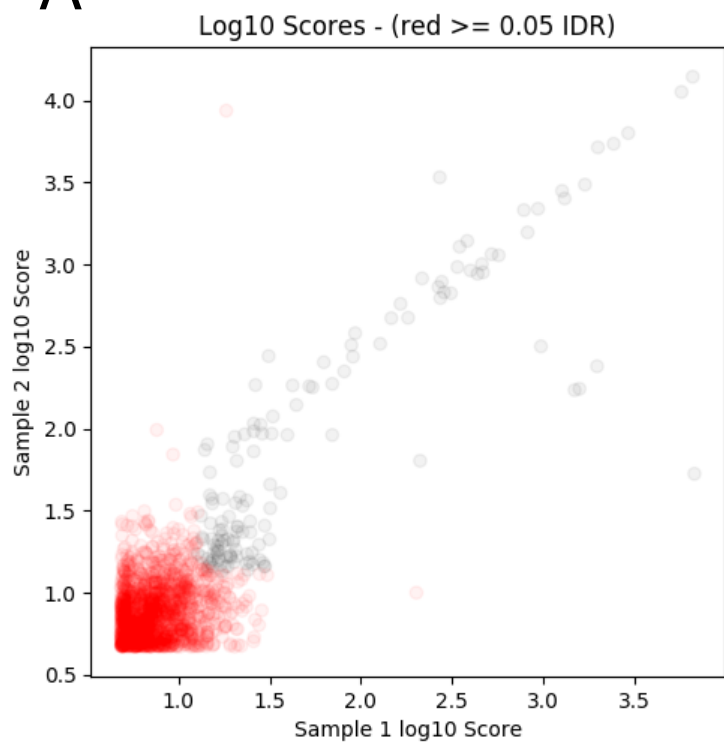**B**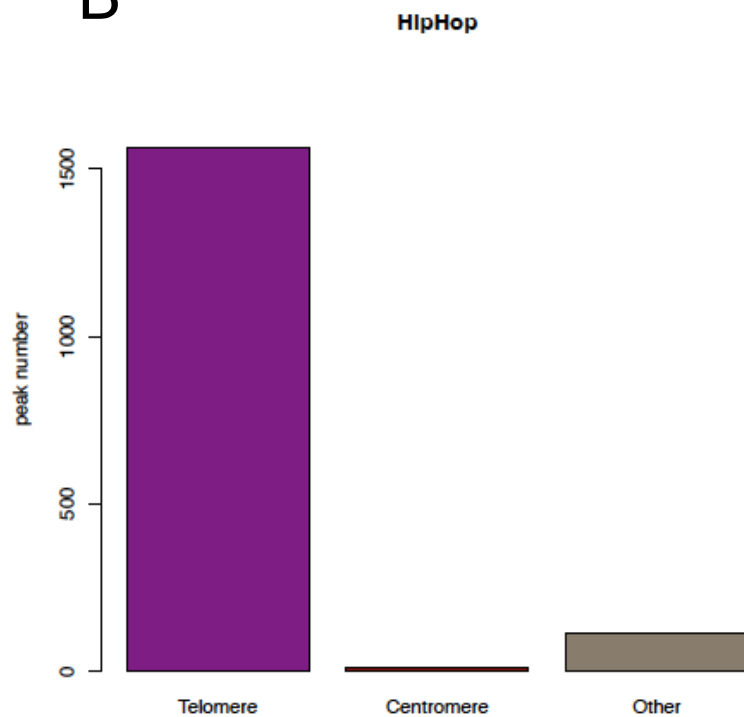**C**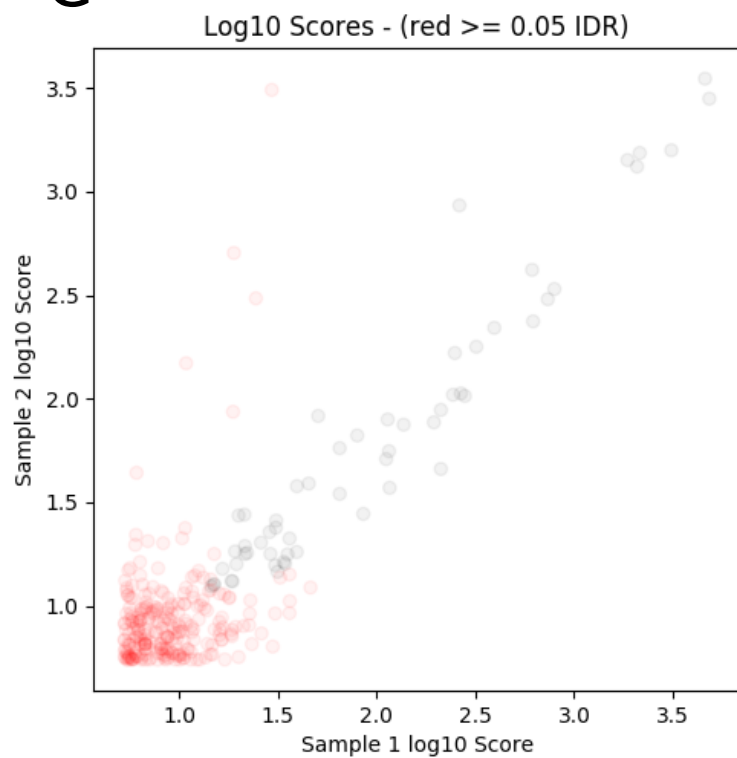**D**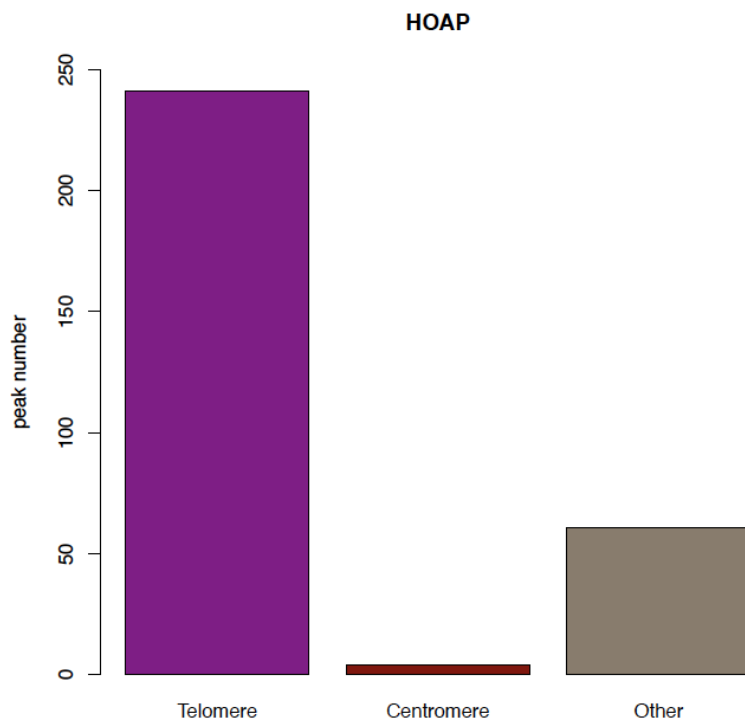

Supplement: S3 Fig — Panels A and C represent the peak scores of replicate 1 versus replicate 2 on a log10 scale. The IDR analyses detected 1686 peaks in common between the two HipHop replicates but only 138 peaks passed the cutoff of IDR<0.05 (in black). The IDR analyses detected 307 peaks in common between the two HOAP replicates but only 58 peaks passed the cutoff of IDR<0.05 (in black). Panels B and D represent the localization of the peaks with an IDR <0.05. The majority of those peaks are localized on telomeres. We also detect a minority of peaks on the centromeres. All the peaks localized outside the telomeres and centromeres are grouped in the category “Other”. However, in this category most of the peaks actually localized on one Y-linked scaffold (Y_scaffold4), which is also enriched in HTT, however this scaffold is unlikely to represent the Y telomere because of its cytological location [37]. (PDF) [file pgen.1010351.s003.pdf]

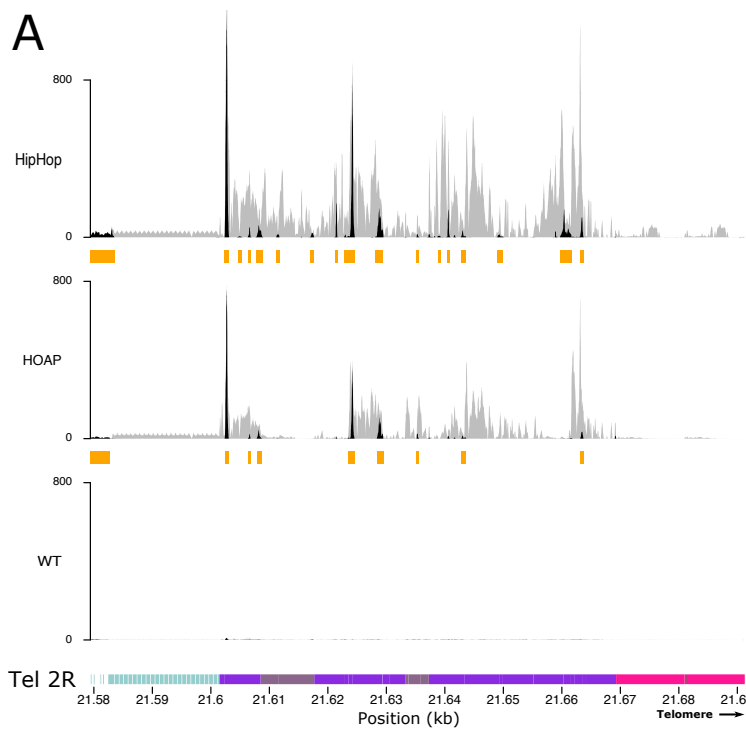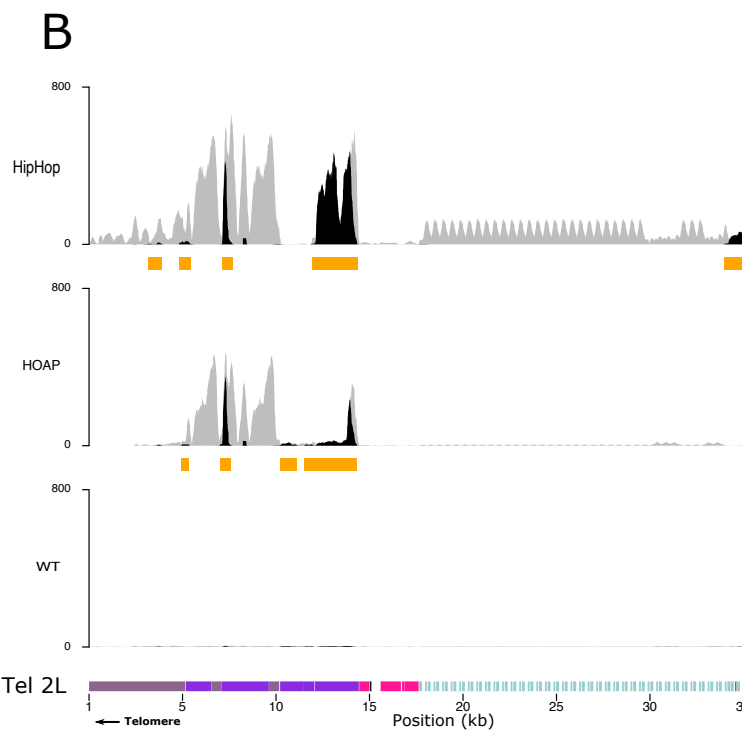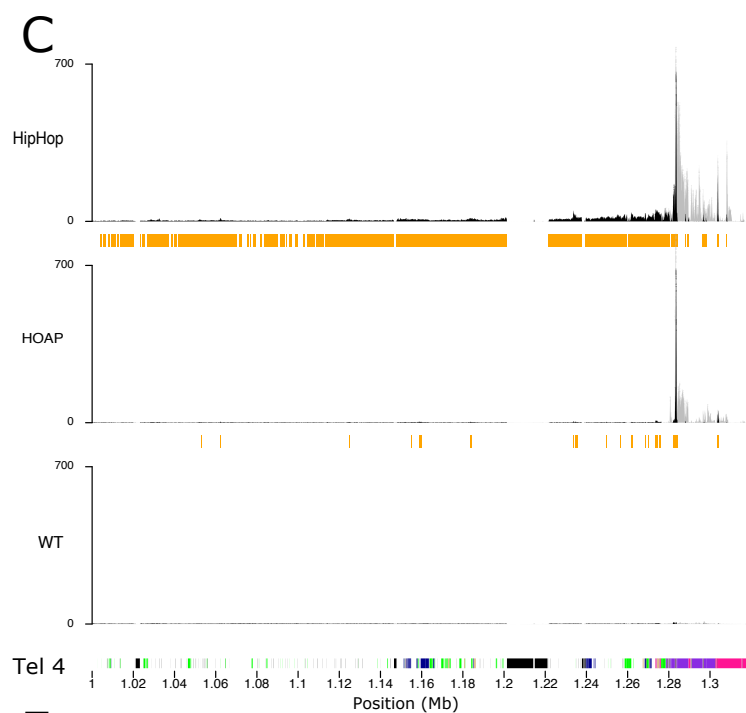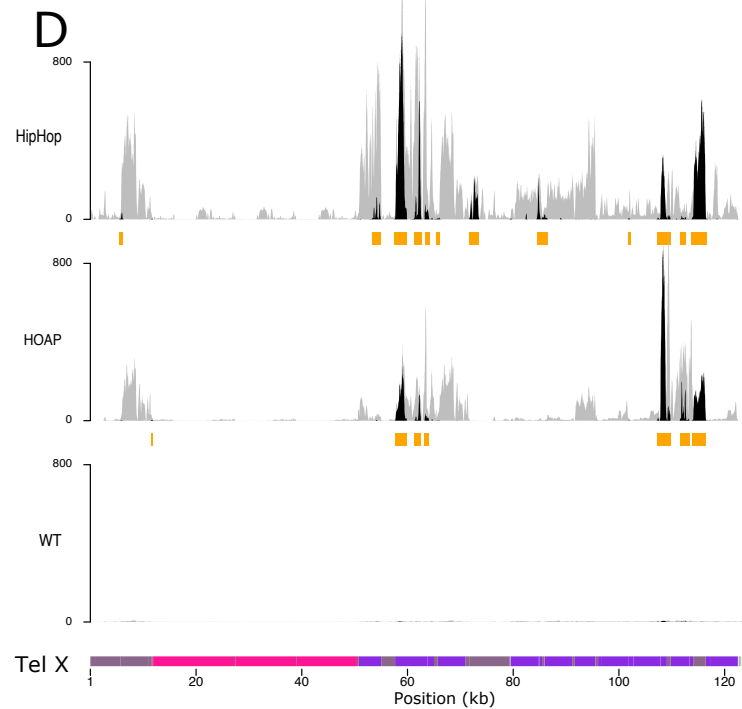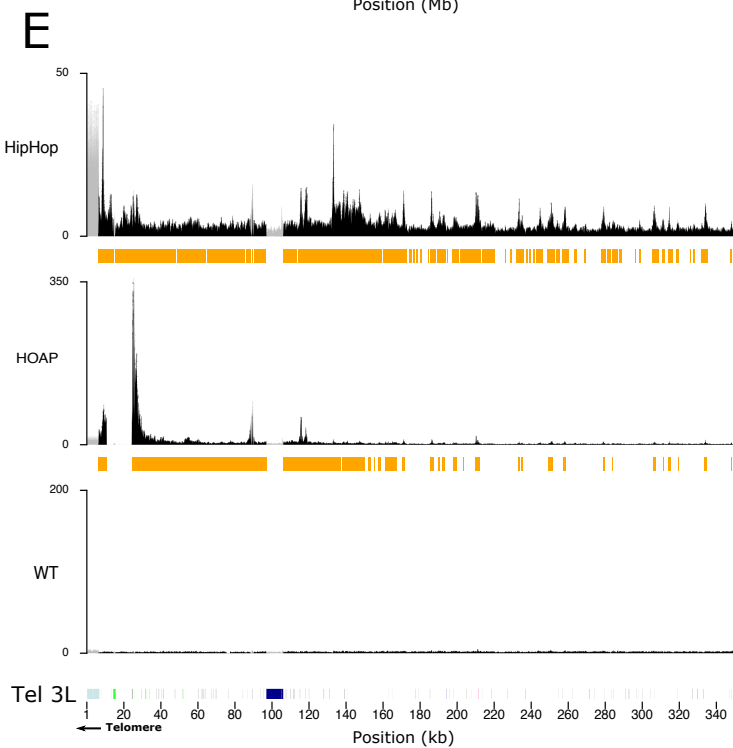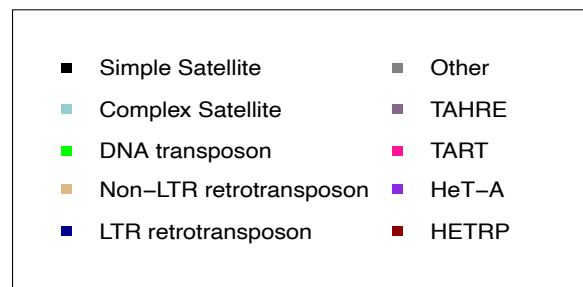

Supplement: S4 Fig — The y-axis represents the normalized enrichment of target protein or the no-tag control (WT) for replicate 1 in RPM. The gray lines correspond to multi-mapped reads, the black lines correspond to the uniquely mapped reads. The orange bars below each plot correspond to MACS2 peaks based on the uniquely mapping reads. The colored cytoband at the bottom of the plot shows the repeat organization. The color code is shown in the legend. (PDF) [file pgen.1010351.s004.pdf]

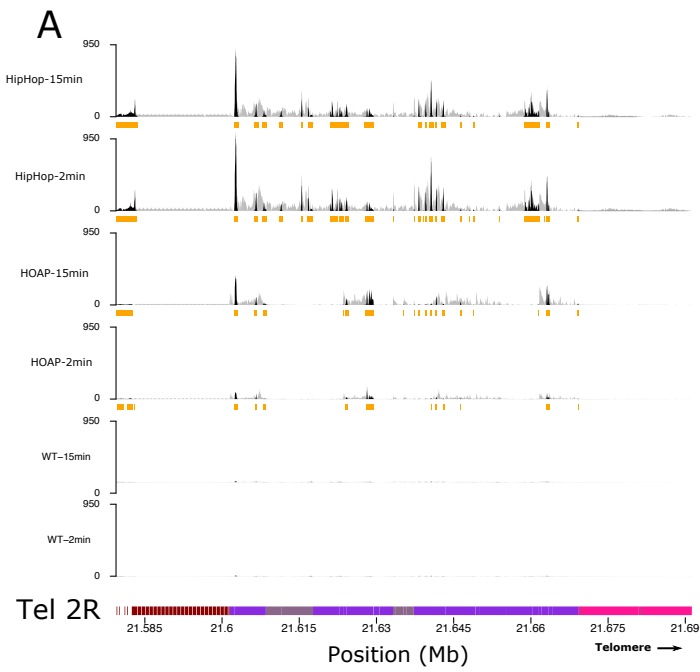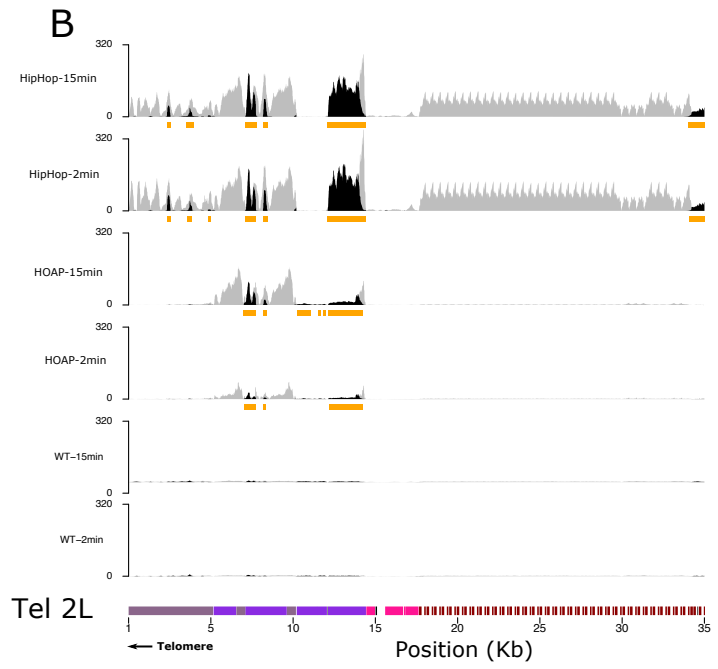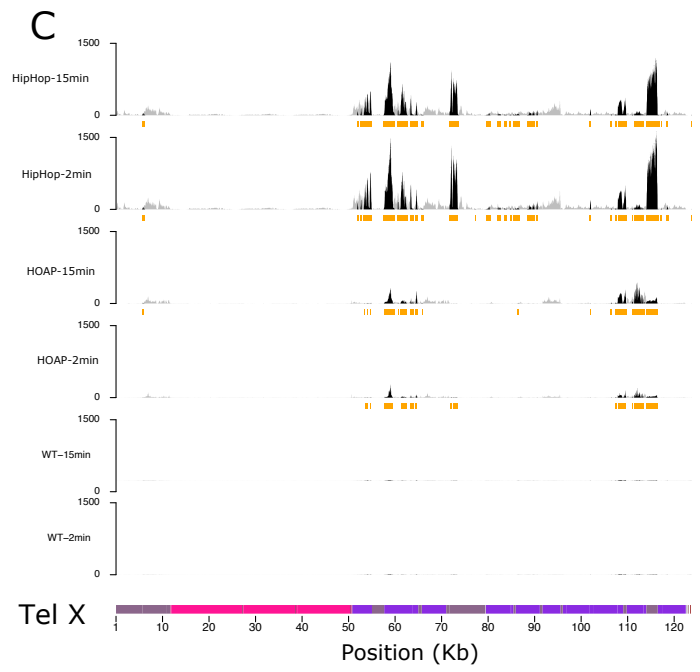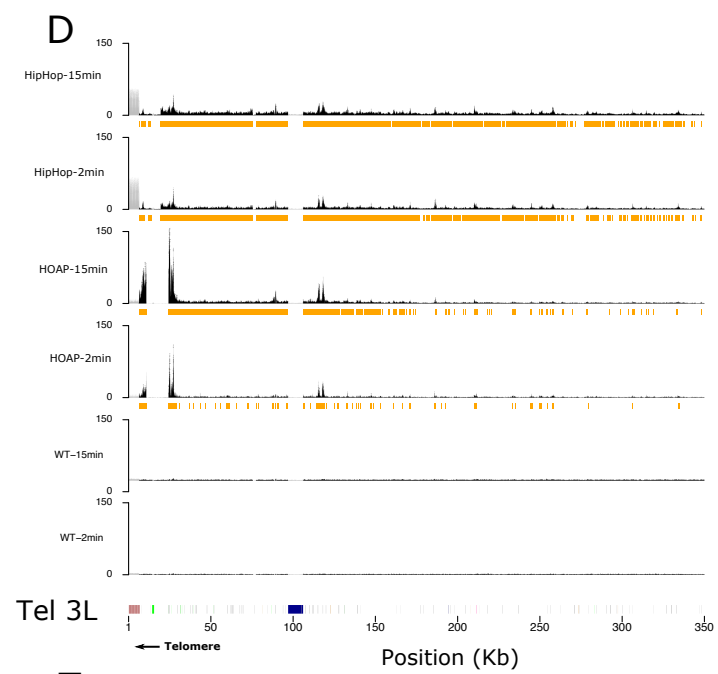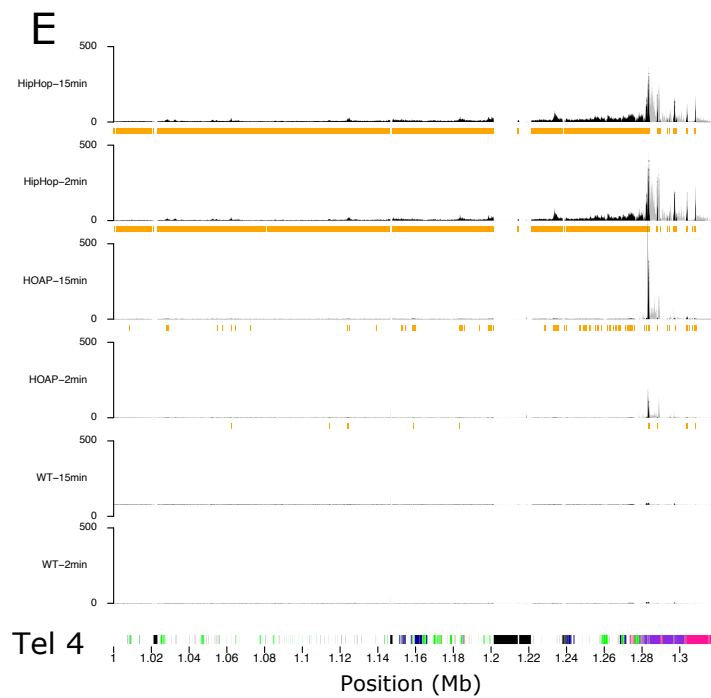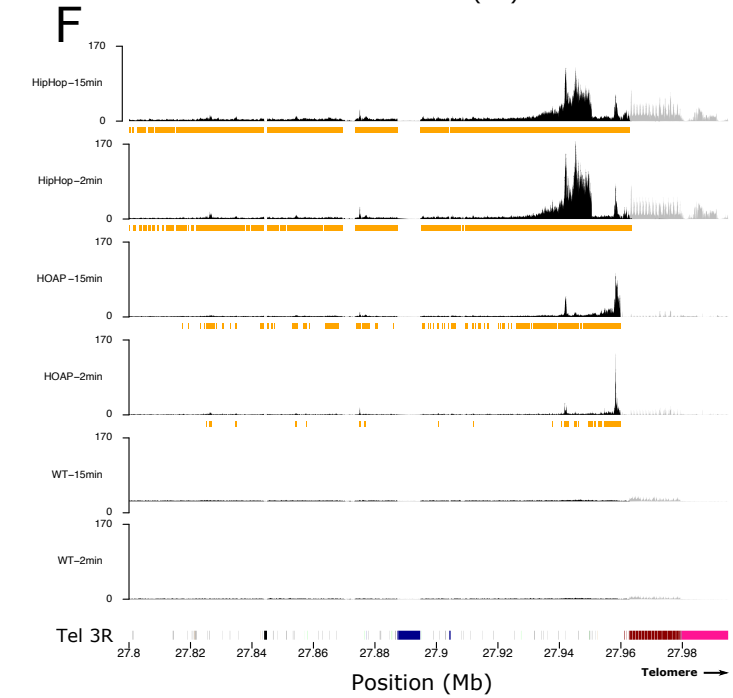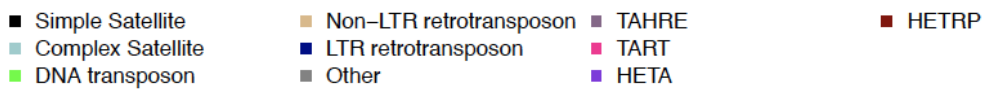

Supplement: S5 Fig — The y-axis represents the normalized enrichment of target protein or the no-tag control (WT) for two different digestion durations (2 or 15 min) in RPM. The gray lines correspond to multi-mapped reads, the black lines correspond to the uniquely mapped reads. The orange bars below each plot correspond to MACS2 peaks based on the uniquely mapping reads. The colored cytoband at the bottom of the plot shows the repeat organization. The color code is shown in the legend. (PDF) [file pgen.1010351.s005.pdf]

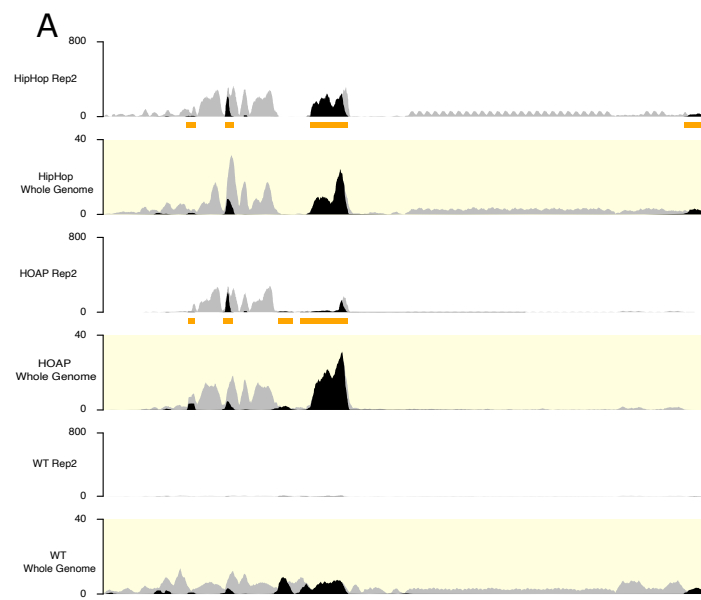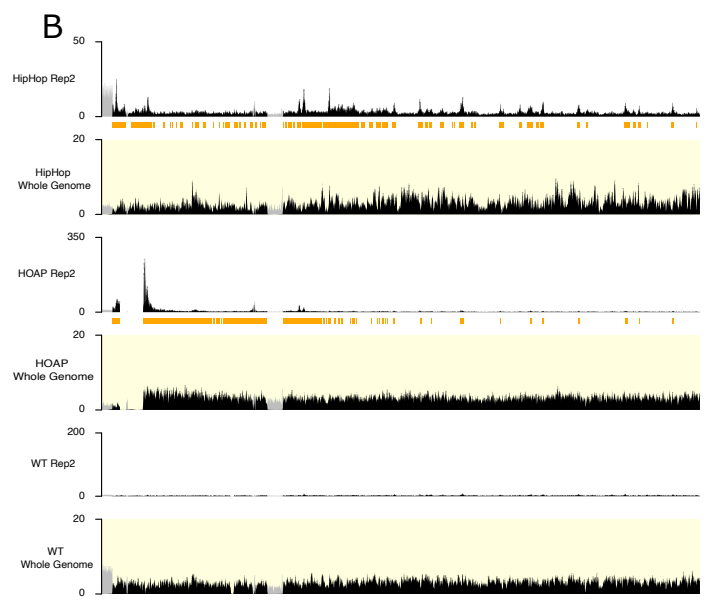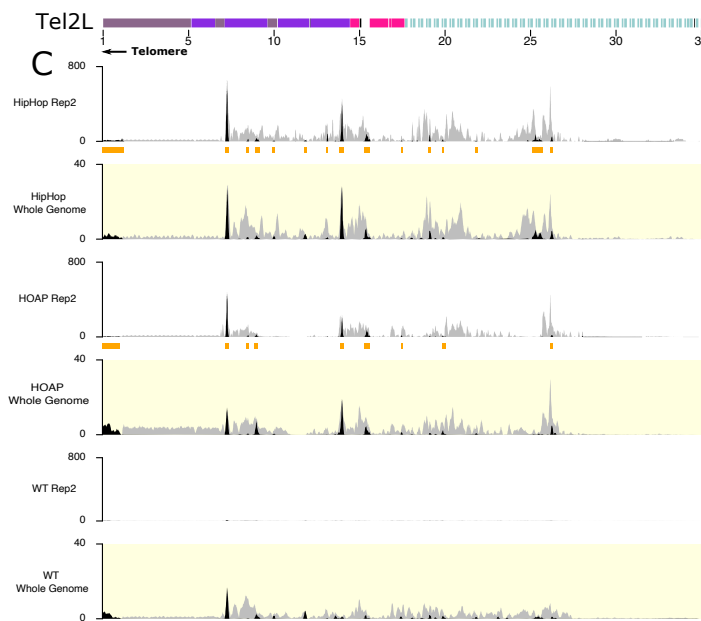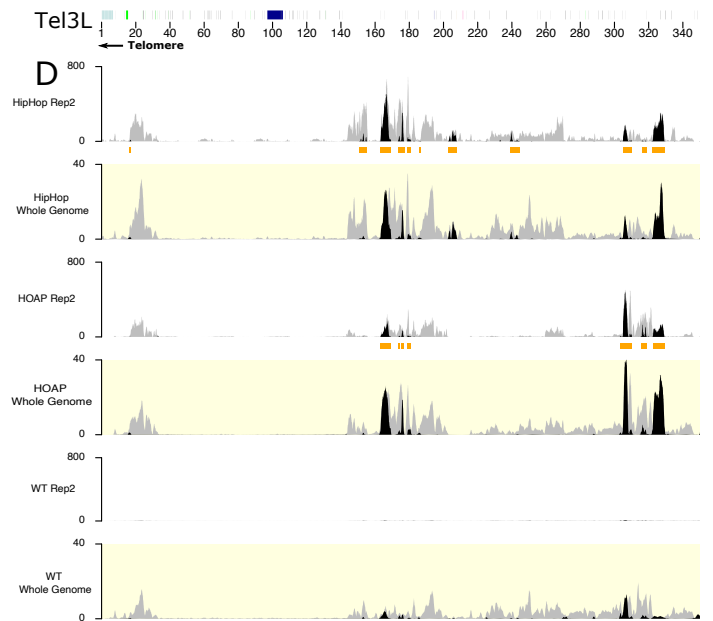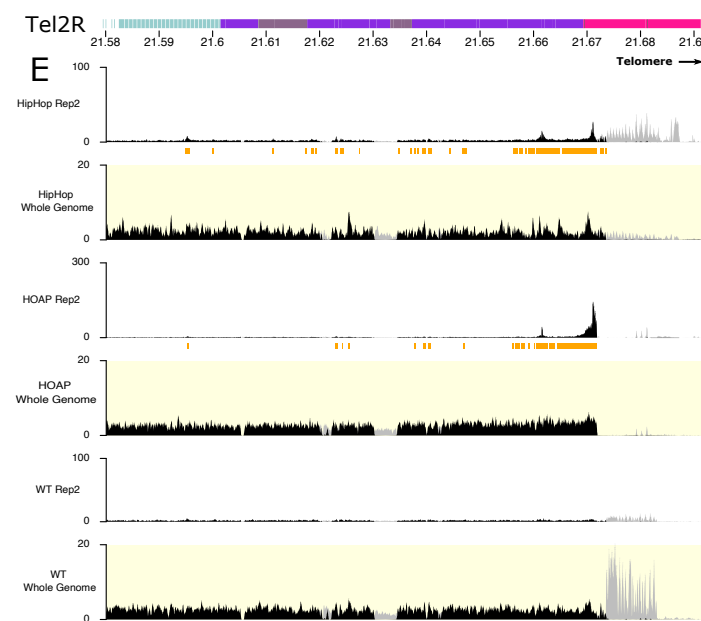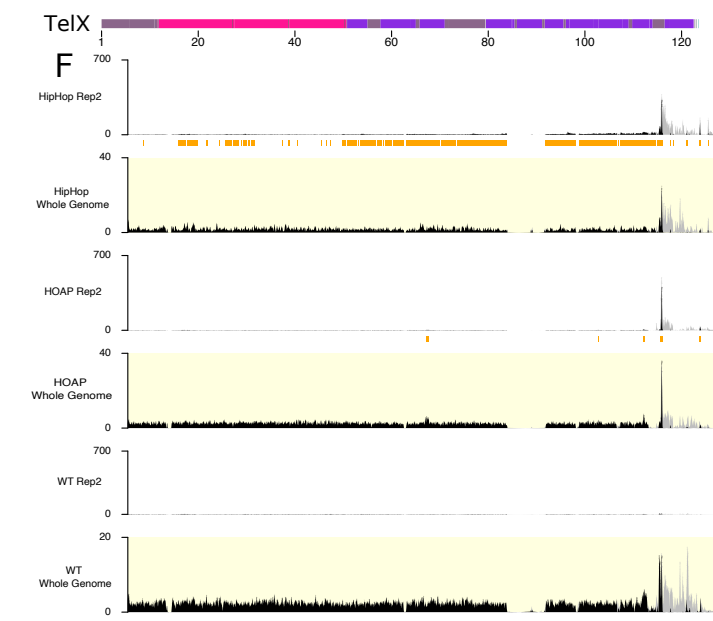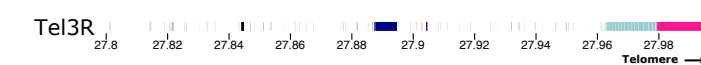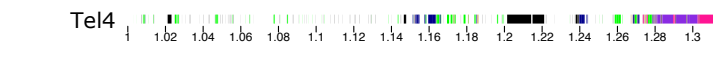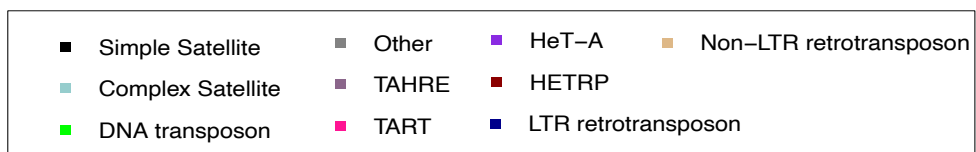

Supplement: S6 Fig — The y-axis of the first plot (white background) represents the normalized enrichment (in RPM) for a second replicate of target protein (HipHop Rep2 and HOAP Rep2) or the no-tag control (WT Rep2). The y-axis of the second plot (yellow background) represents the normalized enrichment (in RPM) of the genomic DNA coverage of each strain (WG: whole genome). The gray lines correspond to multi-mapped reads, the black lines correspond to the uniquely mapped reads. The orange bars below each first plot correspond to MACS2 peaks based on the uniquely mapping reads. The colored cytoband at the bottom of the plot shows the repeat organization. The color code is shown in the legend. (PDF) [file pgen.1010351.s006.pdf]

## HOAP

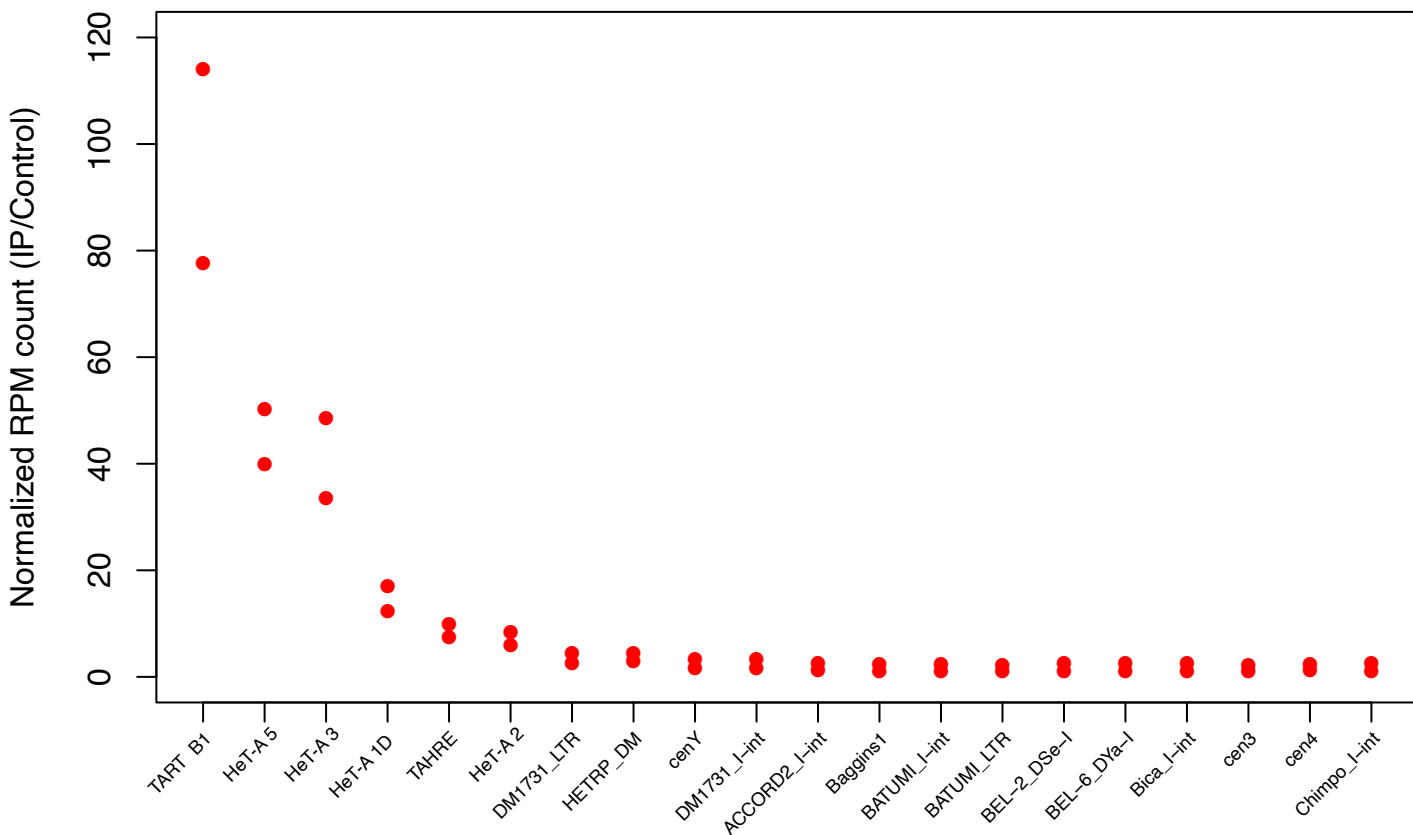

## HipHop

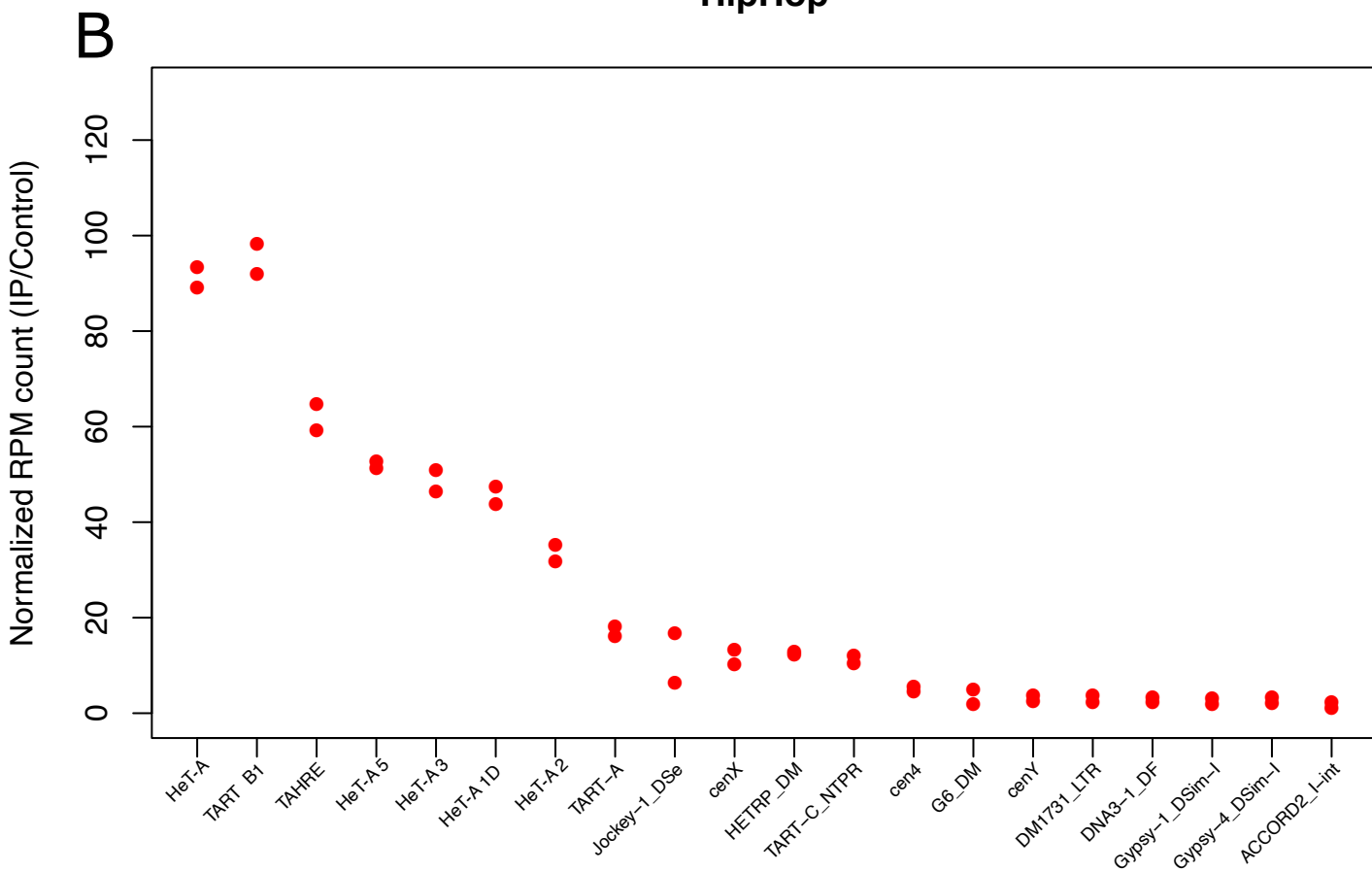

Supplement: S7 Fig — The plot shows the normalized enrichment of target protein over the no-tag control (in RPM) for the top 10 repeats enriched in both HipHop and HOAP nanoCut&Run profiling. The full dataset is in S7 Table. (PDF) [file pgen.1010351.s007.pdf]

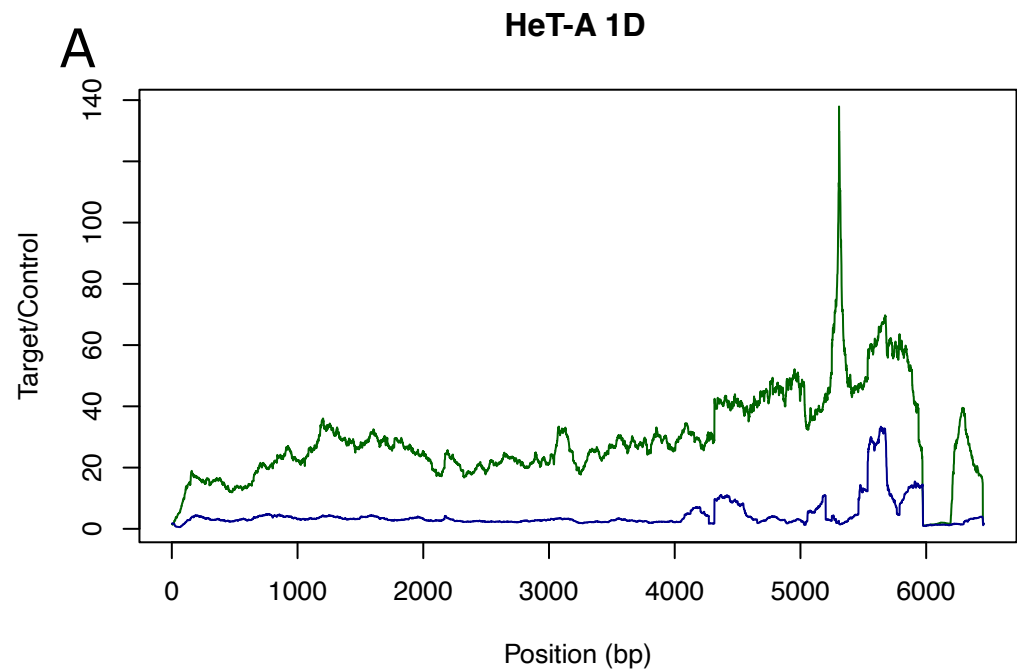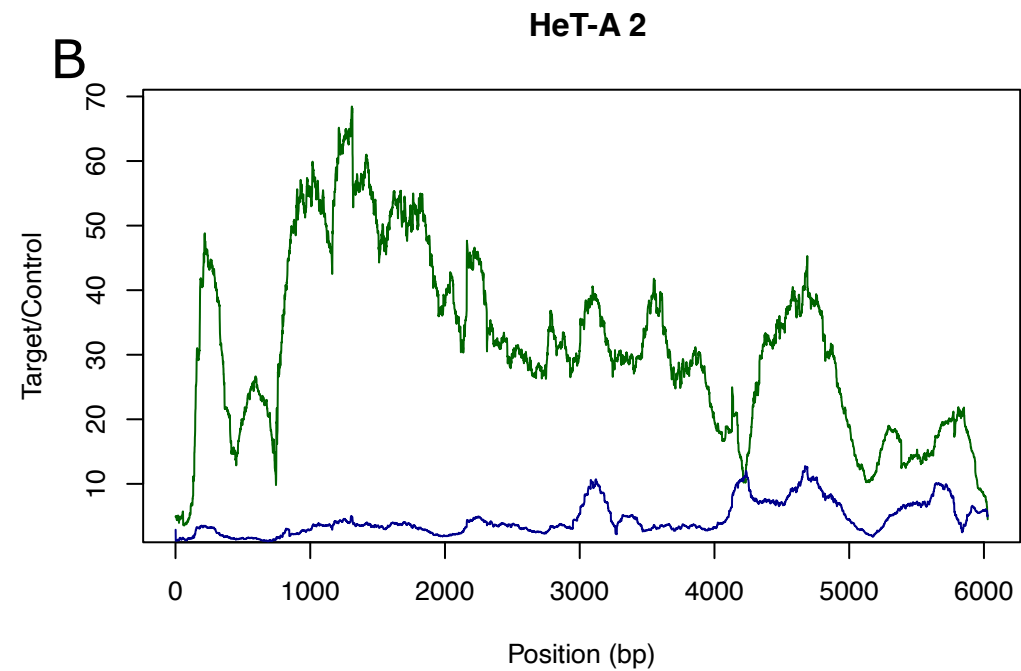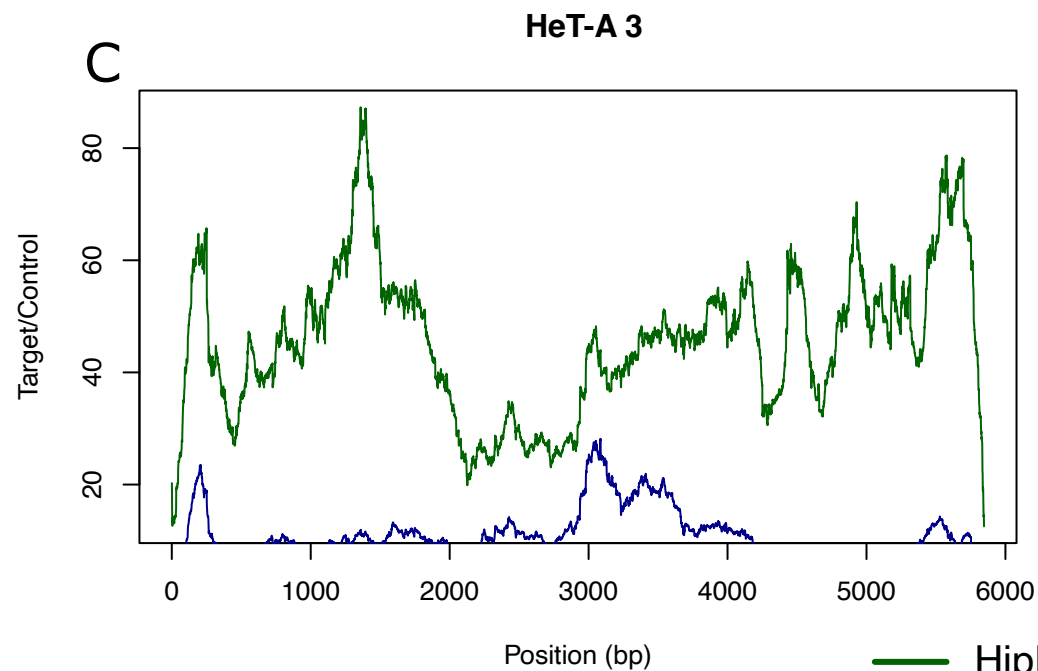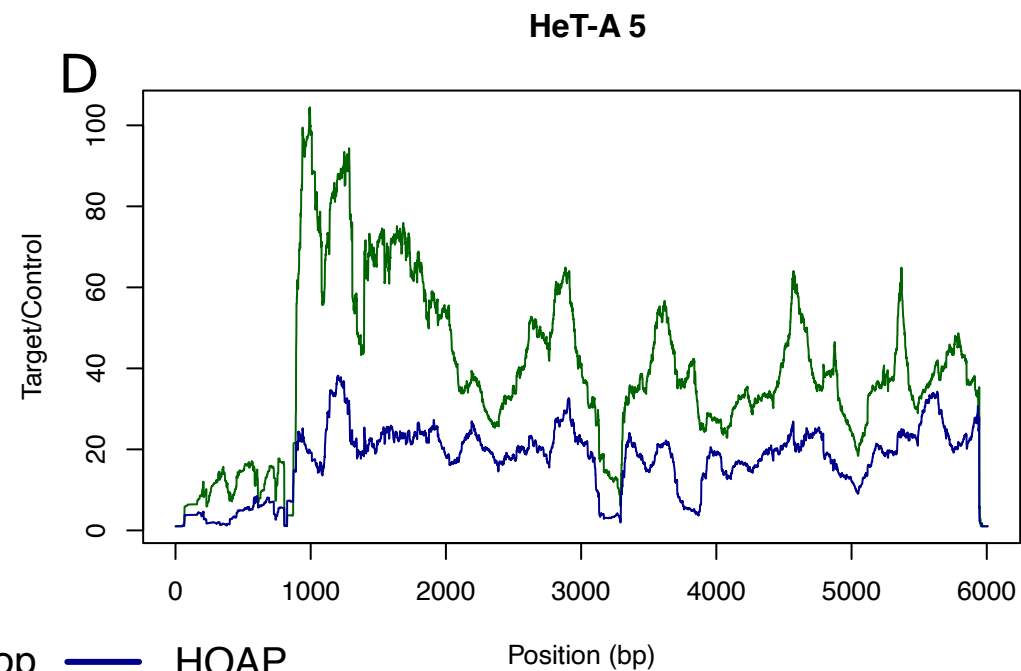

— HipHop — HOAP

Supplement: S8 Fig — Distribution of HipHop and HOAP on the individual subfamilies of HeT-A from [12]. The y-axis represents the mean normalized enrichment (in RPM) of the two replicates for each target protein (HipHop or HOAP) over the no-tag control. (PDF) [file pgen.1010351.s008.pdf]

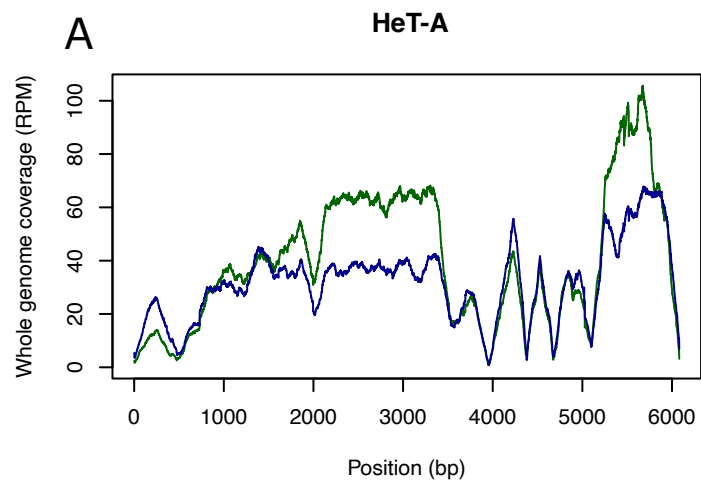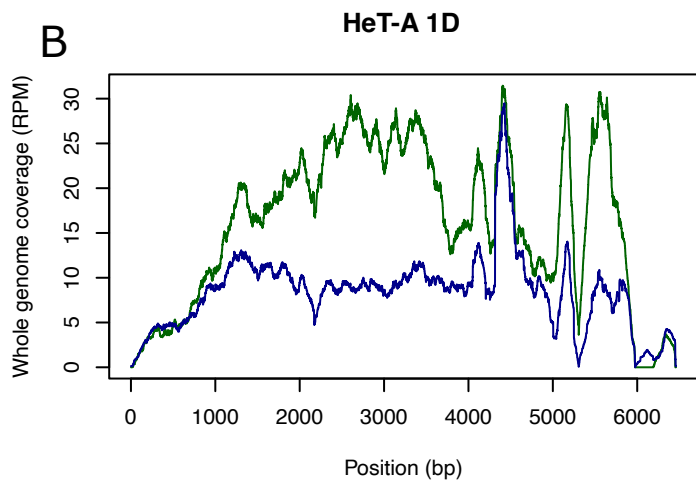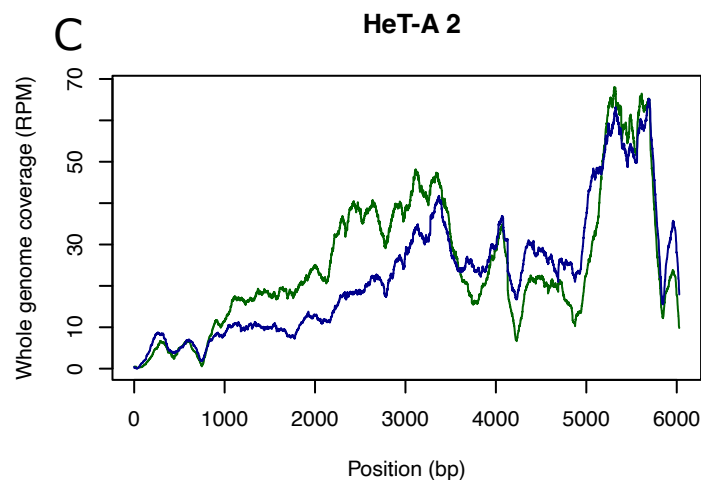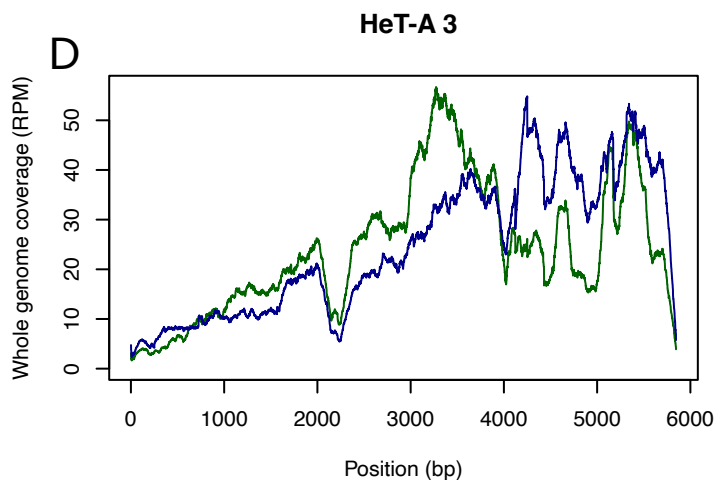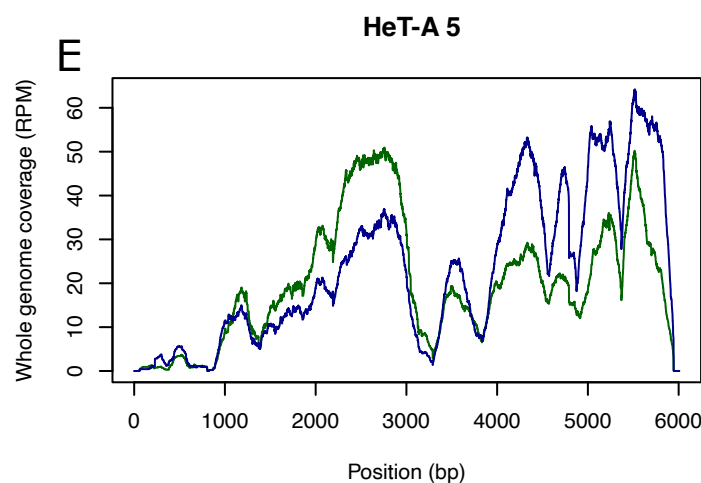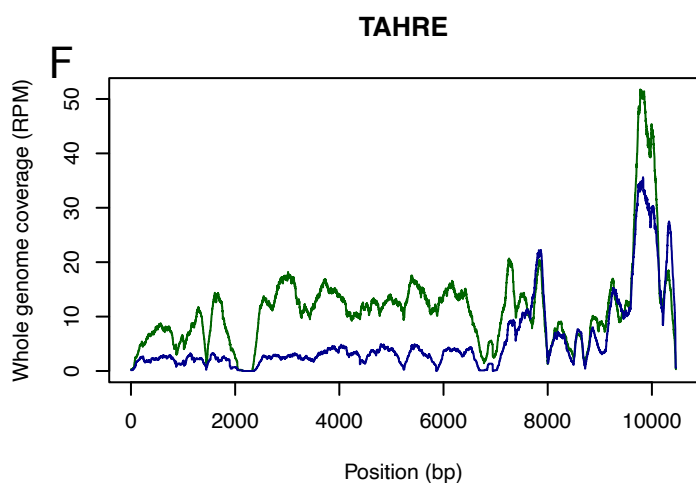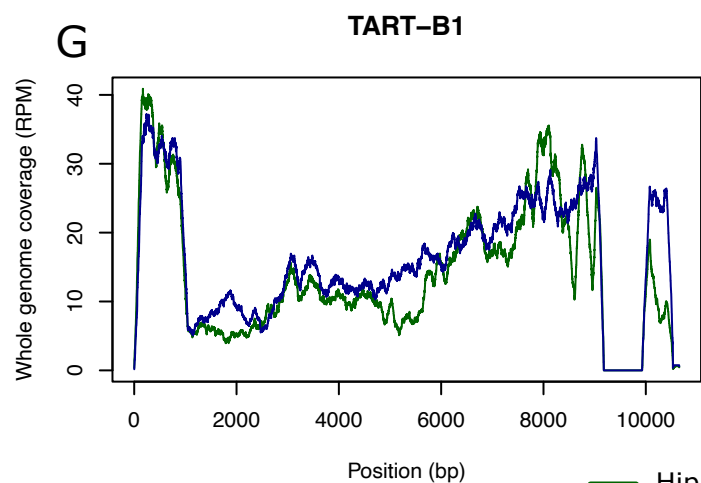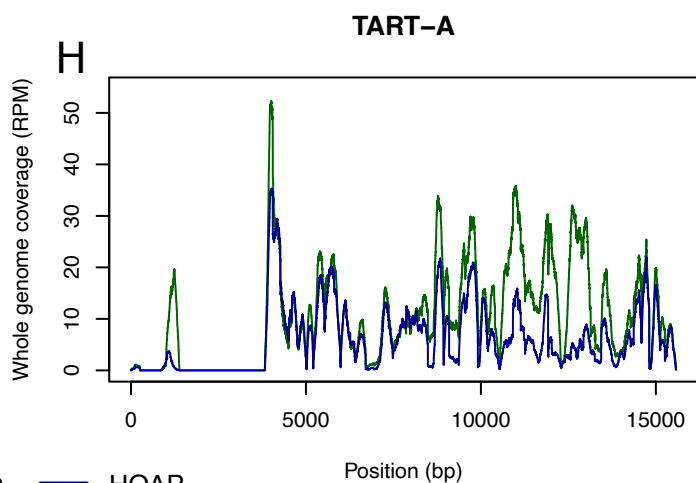

— HipHop — HOAP

Supplement: S9 Fig — Genomic read coverage on HTT elements (TART-A, TART-B, TAHRE, Het-A, Het-A 1D, Het-A 2, Het-A 3, Het-A 5) of GFP-HipHop and GFP-HOAP strains. The y-axis represents the normalized reads coverage in RPM. (PDF) [file pgen.1010351.s009.pdf]

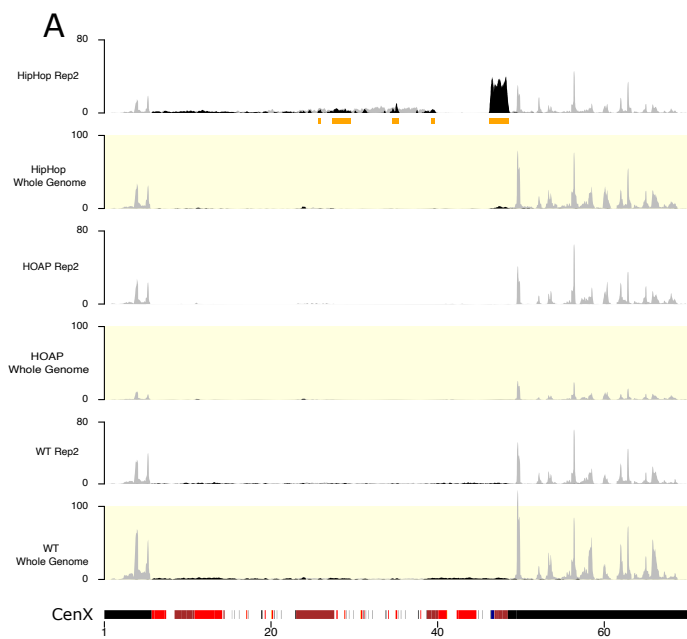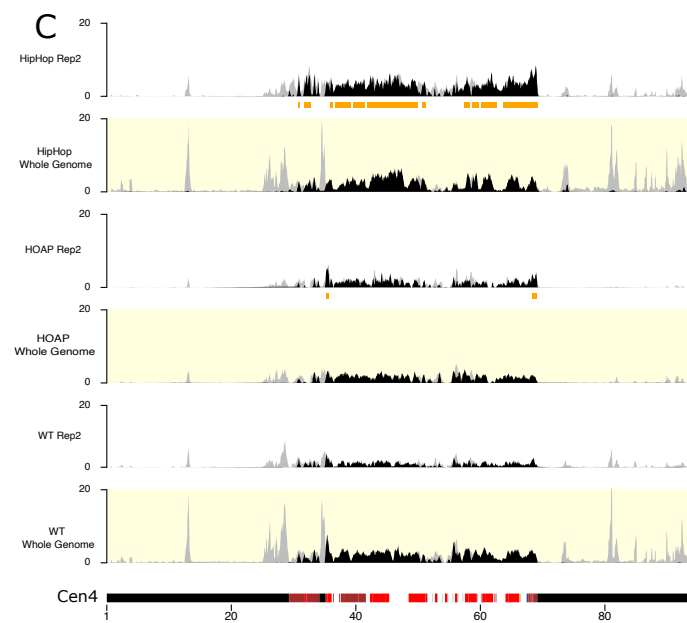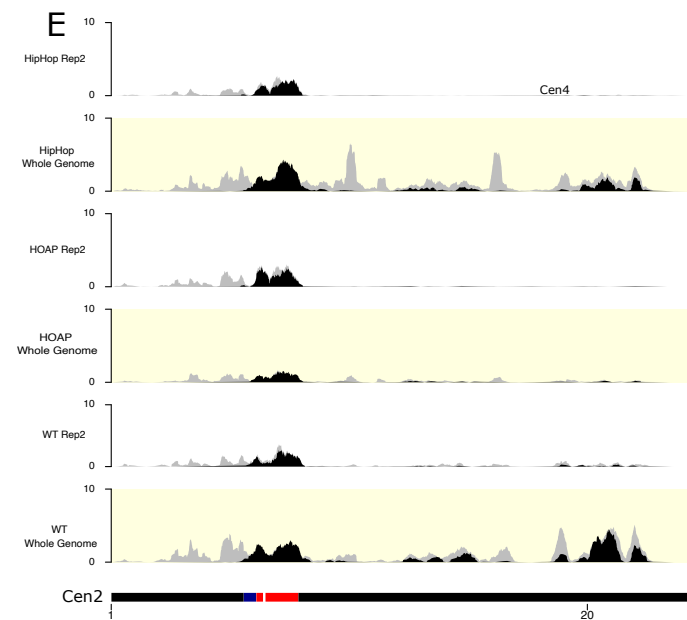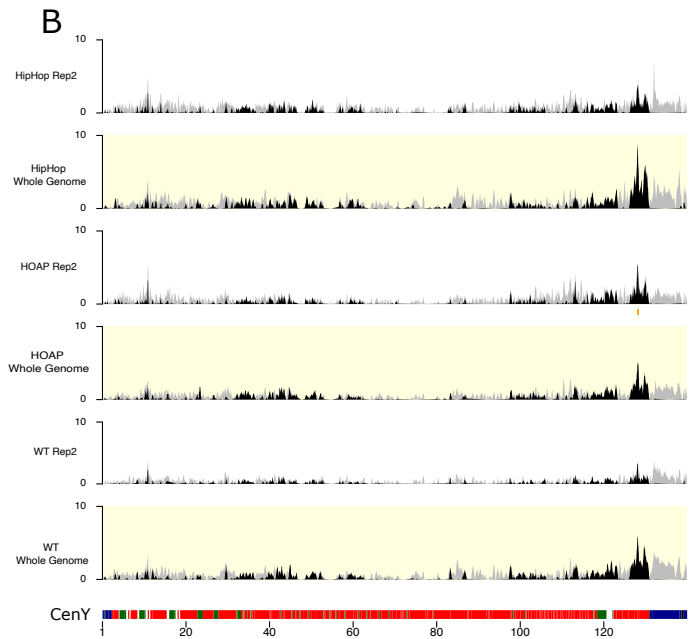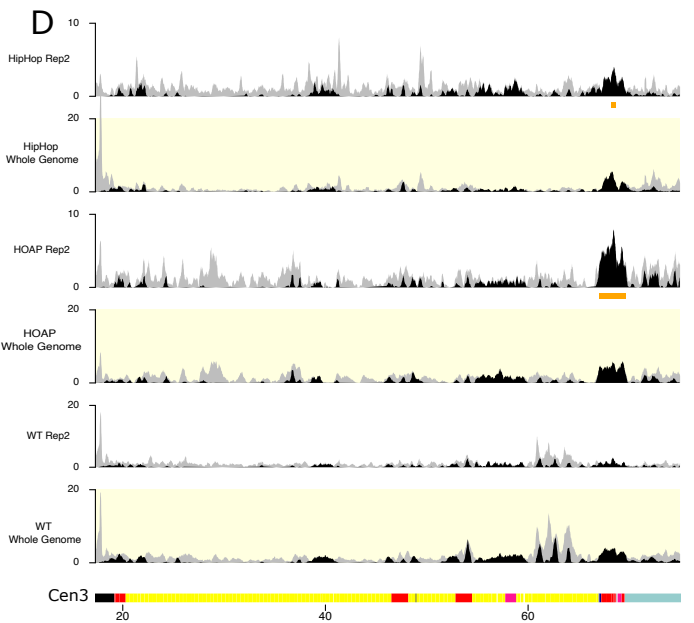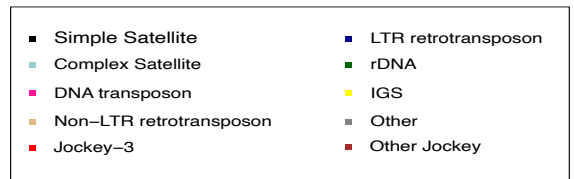

Supplement: S10 Fig — The y-axis of the first plot (white background) represents the normalized enrichment (in RPM) for a second replicate of target protein (HipHop Rep2 and HOAP Rep2) or the no-tag control (WT Rep2). The y-axis of the second plot (yellow background) represents the normalized enrichment (in RPM) of the genomic DNA coverage of each strain (WG: whole genome). The gray lines correspond to multi-mapped reads, the black lines correspond to the uniquely mapped reads. The orange bars below each first plot correspond to MACS2 peaks based on the uniquely mapping reads. The colored cytoband at the bottom of the plot shows the repeat organization. The color code is shown in the legend. (PDF) [file pgen.1010351.s010.pdf]
