# Supplementary material for: The nanoCUT&RUN technique visualizes telomeric chromatin in Drosophila
Source: PLoS Genet. 2022 Sep 1;18(9):e1010351. doi: 10.1371/journal.pgen.1010351 (PMC9473618; doi:10.1371/journal.pgen.1010351)
Supplement: S1 Protocol — (DOCX) [file pgen.1010351.s019.docx]

**nanoCut&Run Protocol**

Starting from purified nuclei

**Nuclei Binding to Beads**

(1) Gently resuspend Bio-Mag Plus Concanavalin A coated beads before use,

(2) Withdraw about 20μl beads in slurry for each sample, and transfer into a 1.5 ml Eppendorf tube containing 1 ml of binding buffer, mix gently with pipette,

(3) Place the tube on a magnetic stand and let clear for 1 min, pipette off the liquid, wash twice with 1 ml binding buffer at RT,

(4) Resuspend the beads in 300 μl of binding buffer, swirl the purified nuclei gently before slowing adding the beads, and rotate to mix at RT for 5 min.

**Nuclei Blocking**

(5) Place the tube on magnetic stand, clear for 1 min and gently remove the liquid,

(6) Add 1ml of WBSED buffer, rotate at RT for 5 min.

**nanoGM Binding**

(7) Pipette off WBSED buffer, replace with nanoGM dilution (250 ng in 1 ml WBSED), incubate at 4°C for 2 h,

(8) Place the tube on the magnetic stand, clear for 1 min and pull off the liquid, wash twice with 1 ml WBSED buffer.

**nanoGM Digestion**

(9) Pipette off the liquid, resuspend with 150 μl prechilled WBC digestion buffer, incubate at 4°C for different time lengths,

(10) Stop the reaction at each time point with 200 μl STOP buffer, mix by inversion, incubate at 4°C for 2 min,

(11) Incubate the tube at 37°C for 30 min, Centrifuge for 5 min at 16000g, transfer the supernatant to a new 1.5 ml Eppendorf tube.

**DNA extraction**

(12) Add 3.5 μl of 10% SDS and 2.5 μl of Proteinase K (20 mg/ml), mix by inversion, incubate at 70°C for 10 min,

(13) Add 300 μl phenol-chloroform-isoamyl solution (25:24:1), vortex for 30 s,

(14) Centrifuge MaXtract phase lock tubes with 15,000g for 1 min at 4°C in advance,

(15) Transferred the mixture from step #11 to the MaXtract phase lock tube, and centrifuge for 5 min at 16000g,

(16) Transfer the aqueous layer into a new 1.5 ml Eppendorf tube, then add 300 μl of chloroform, vortex for 30 s,

(17) Transferred the mixture to new MaXtract phase lock tubes, centrifuge for 5 min at 16000g,

(18) Transferred the aqueous layer into a new 1.5 ml Eppendorf tube, add 150 μl of AMPure XP beads, mix gently, let it sit at RT for 10 min,

(19) Placed the sample on a magnetic stand, clear for 2 min, Transfer supernatant to a new Eppendorf tube,

(20) add 1 ml 100% ethanol, mix by pipetting gently,

(21) Store at -20°C overnight, after centrifugation for 10 min at 16000g, discard the supernatant,

(22) After the pellet dries, dissolve with 20 μl ddH2O.

**Reagent preparation**

WBE buffer (50 ml)

| Volume | Ingredient | Final concentration |
| --- | --- | --- |
| 1 ml | 1 M HEPES pH7.5 | 20 mM |
| 1.5 ml | 5 M NaCl | 150 mM |
| 166 μL | 30% BSA | 0.1% |
| 200 μL | 0.5 M EDTA | 2 mM |
| 47.5 ml | ddH2O |  |
|  |  |  |

WBSED buffer (4 ml/sample, 12 ml)

| Volume | Ingredient | Final concentration |
| --- | --- | --- |
| 12 ml | WBE |  |
| 30 μL | 200 mM spermdine | 0.5 mM |
| 120 μL | 5% digitonin | 0.05% |
| 1 | small Roche complete EDTA-free protease inhibitor tablet | |

Binding buffer (20 ml)

| Volume | Ingredient | Final concentration |
| --- | --- | --- |
| 400 μL | 1 M HEPES-KOH pH 7.9 | 20 mM |
| 200 μL | 1 M KCl | 10 mM |
| 20 μL | 1 M CaCl2 | 1 mM |
| 20 μL | 1M MnCl2 | 1 mM |
| 19.4 ml | ddH2O |  |

WBC digestion buffer (10 ml)

| Volume | Ingredient | Final concentration |
| --- | --- | --- |
| 200 μL | 1 M HEPES pH 7.5 | 20 mM |
| 300 μL | 5 M NaCl | 150 mM |
| 34 μL | 30% BSA | 0.1% |
| 120 μL | 1 M CaCl2 | 12 mM |
| 9.5 ml | ddH2O |  |

STOP buffer (5 ml)

| Volume | Ingredient | Final concentration |
| --- | --- | --- |
| 340 μL | 5 M NaCl | 340 mM |
| 200 μL | 0.5 M EDTA | 20 mM |
| 100 μL | 0.2 M EGTA | 4 mM |
| 25 μL | 10 mg/ml RNase A | 50 μg/ml |
| 10 μL | 20 mg/ml glycogen | 40 μg/ml |

**Key Reagents and Materials:**

Bio-Mag Plus Concanavalin A (Polysciences, 86057)

Spermidine (Sigma, S0266-5G)

AMPure XP Magnetic Beads (Beckman Coulter, A63882)

MaXtract phase-lock microcentrifuge tubes (Qiagen, 129046)

small Roche complete EDTA-free protease inhibitor tablet (Roche, 04693132001)

Glycogen (Invitrogen, R0561)

Ribonuclease A (TaKaRa, 2158)
