# Supplementary material for: The nanoCUT&RUN technique visualizes telomeric chromatin in Drosophila
Source: PLoS Genet. 2022 Sep 1;18(9):e1010351. doi: 10.1371/journal.pgen.1010351 (PMC9473618; doi:10.1371/journal.pgen.1010351)
Supplement: S2 Protocol — (DOCX) [file pgen.1010351.s020.docx]

**Expression and Purification of nGFPMNase:**

1. nGFPMNase fusion protein was overexpressed in *E.coli* BL21(DE3) Condon Plus transformed with a pET28a based vector (Addgene ID: 187826) and purified by His affinity chromatography.
2. 100 μl of pre-cultured bacteria was inoculated into 100 ml of LB medium, incubated under the conditions of 37℃ and 220 rpm until an OD600 (0.6 to 0.8) was reached.
3. Protein expression was induced with IPTG at a final concentration of 0.5 mM, and lasted for 3 to 4 hours. The culture was pelleted by 13000g and 4℃ for 10 min. The bacteria pellet was resuspended in Lysis buffer, and sonicated for 30 min (5s on /5s off). Lysed cells were centrifuged at 13000g and 4℃ for 10 min. Supernatants were collected.
4. Supernatants were incubated with 1ml Ni-NTA beads for 1 h at room temperature, transferred to a purification column that had been pre-equilibrated with 10 ml of lysis buffer.
5. The Ni-NTA beads were collected and successively washed with ten bed volumes of wash buffer I and wash buffer II .
6. nGFPMNase fusion protein was eluted with 3 ml of Elution buffer, and desalted with a desalting column per manufacturer’s instruction. Purified nGFPMNase fusion protein was stored in 10 mM Tris-HCl buffer.
7. Reagent preparation

7.1 LB medium (1 L pH 7.0): 10 g of Tryptone, 10 g of NaCl, 5 g of Yeast extract

7.2. Lysis buffer (50 ml pH 8.0)

Quantity Ingredient Final concentration

0.35 g Na2HPO4 20 mM

0.88 g NaCl 0.3 M

half of a of complete EDTA-free protease inhibitor tablet

7.3. Wash buffer I (50 ml pH 8.0)

Quantity Ingredient Final concentration

0.35 g Na2HPO4 20 mM

1.46 g NaCl 0.5 M

7.4. Wash buffer II (50 ml pH 8.0)

Quantity Ingredient Final concentration

0.35 g Na2HP04 20 mM

1.46 g NaCl 0.5 M

0.034 g Imidazole 10 mM

7.5. Elution buffer (50 ml pH 7.5)

Quantity Ingredient Final concentration

0.12 g Tris 20 mM

0.29 g NaCl 100 mM

0.85 g imidazole 250 mM

NI-NTA beads (QIAGEN, 30210)

GE PD-10 Desalting Columns (GE healthcare, 17-0851-01)
