# Supplementary material for: The nanoCUT&RUN technique visualizes telomeric chromatin in Drosophila
Source: PLoS Genet. 2022 Sep 1;18(9):e1010351. doi: 10.1371/journal.pgen.1010351 (PMC9473618; doi:10.1371/journal.pgen.1010351)
Supplement: Attachment — Submitted filename: AuthorResponsesToReviewersComments.docx [file pgen.1010351.s021.docx]

Reviewer #1

***Reviewer:*** *The manuscript entitled “The nanoCUT&RUN technique visualizes telomeric chromatin in Drosophila” by Chen and colleagues describes work from a collaborative team focused on applying a modified CUT&RUN technique to investigate Drosophila telomere chromatin. The authors developed a GFP-nanobody based modified CUT&RUN method and use this new method to initially profile TRL as a positive control. Then, they apply the new method to the telomere capping proteins HipHop and HOAP, which are confirmed present at the telomeres. Interestingly, they find HipHop also present at the centromere.*

*The modified CUT&RUN method will be of use to individuals interested in profiling GFP-tagged proteins. In particular, if specific isoforms are tagged, this method is useful, as often isoforms cannot be distinguished by protein antibodies. However, it would also seem that one can achieve the same goal by using a GFP-tagged protein and an anti-GFP antibody in the regular CUT&RUN protocol.*

**Authors:** We agreed with the reviewer that an anti-GFP antibody could potentially be used to achieve the same goal. However, we wish to make the following arguments: (1) for polyclonal anti-GFP antibodies, one would have to experiment with different conditions with every purified batch; (2) for monoclonal antibodies (similar to a nanobody) this would not be necessary so in theory it would serve the purpose. In practice , however, our collective experiences have been that a well characterized anti-GFP antibody with consistent performance in IP-related experiments has not been widely available; (3) on the other hand, our nanobody, expressed in bacteria as a direct fusion to MNase, would offer a platform for comparing results from different experimental setups. We added a discussion **on pages 5-6** of the revision to raise these points. It reads: “In our studies of Drosophila chromatin proteins, we often employed tagging with a Green Fluorescent Protein (GFP) [e.g. Schneiderman et al. 2012; Wesolowska et al. 2013; Tang et al. 2017; Huang et al. 2021] so that live imaging of the target proteins could be achieved as long as the GFP-tagged target had been proven functional in genetic rescuing experiments. In theory, an anti-GFP antibody could be used to conduct Cut&Run profiling of any such target. However, our collective experiences with monoclonal anti-GFP antibodies have been unsatisfactory. Since single-domain anti-GFP nanobodies offer consistent performance in IP related experiments [e.g. Cicconi et al. 2017; Donohoe et al. 2018], and can be readily purified from bacteria, we were prompted to develop a method generally applicable to profiling GFP-tagged chromatin proteins .”

***Reviewer:*** *Overall, the manuscript is well written, the figures are clearly laid out, and the data provided support the conclusions drawn. I have several comments and questions regarding the analyses and experiments carried out that should be addressed to improve the manuscript and allow interested parties to take advantage of the new nanoCUT&RUN method.*

*•	In several places in the manuscript, data are described as being consistent with each other (replicates, short versus long TRL isoform, 2min vs. 15min digestion samples, etc) or with reported data from ChIP. Sometimes, correlation coefficients are reported, but not always, and statistical test and p-values for the comparisons are missing. These should be reported to allow the reader to evaluate the strength of the correlation.*

**Authors:** We added info about statistical tests and presented the p-values generated from the tests.

***Reviewer:*** *•	What is the evidence that the lower correlation between the two datasets for the long TRL isoform is due to over-digestion? If there was an issue with sample quality, why was the experiment not repeated?*

**Authors:** We have deleted our assessment about over digestion. There was not an issue with sample quality. Since the TRL trials successfully showed the feasibility of the method, we did not repeat the experiment but decided to spend our efforts on the telomeric aspect of the study.

***Reviewer:*** *•	For the HipHop and HOAP experiments, biological replicates are lacking, and the conclusions presented are derived from one replicate each of two different experimental protocols (2 min. vs. 15 min. digestion). The lack of replication is concerning, as typically at least 3 biological replicates are required for genomics experiments. Also, from the methods section, it is not clear if the 2 min and 15 minute datasets represent two different biological samples or are derived from the same pool of nuclei. It is also not clear why data from 2 min. digestion and 15 min. digestion were collected.*

**Authors:** We agree that replication of the experiments would improve the scientific rigor of our study, and have completed two more biological replicates for the HipHop and HOAP experiments. The new data continue to support our main conclusions. We chose a 2 min and a 15 min time point after having estimated the reasonable amount of time needed to complete two rounds of handling multiple samples.

***Reviewer:*** *•	The authors report a lower level of enrichment for HOAP at the telomere than for HipHop. Is this observation consistent with data obtained from other experimental methods (ChIP, polytene chromosome analysis)?*

*If so, given that similar numbers of sequence reads were obtained for HOAP and HipHop, where do all the additional reads map to, if they are not at the telomere?*

**Authors:** Such a comparison has not been made in prior studies, and it would have been problematic since the efficacy of antibodies against different antigens has not been compared and would be difficult to do so. On the other hand, since the same GFP nanobody was used in our profiling experiments, such a comparison is possible. There are no particularly enriched regions of the genome outside of the telomere and centromere, that is, there is no specific pattern. We see that for these “off target” locations, both HipHop and HOAP are enriched.

***Reviewer:*** *•	For the TART-B analysis, the HOAP 15 minute experiment is very different from the 2 minute experiment, while for all other analyses, the two types of digestions result in very similar patterns. Is this really different, and if so, why?*

**Authors:** Other repeats also show a difference between the two HOAP treatments (e.g. Het-A_3 and Het-A_5)). Based on the new replicates, these differences appear to be driven by a technical bias.

***Reviewer:*** *•	Based on their mapping of HOAP and HipHop to the consensus telomere TEs, the authors conclude that the two proteins have not sequence specificity in their binding. In my opinion that conclusion is not entirely supported by the data. I understand that the authors argue that the variability in mapping across the TE is likely due to sequence variability across the diverse TE copies. However, these data cannot distinguish between enrichment variability due to binding preference or due to a bias in the consensus TE sequence used.*

**Authors:** We agree that the appearance of a lack of preferred binding sites for the telomeric proteins could actually be due to a bias in the consensus sequence of telomeric elements. We wish to point out that motif analysis on actual HipHop cut&run reads did not reveal any candidates for preferred binding sites. **On page 13**, it reads: “Consistently, we did not uncover any particularly strong motif(s) enriched among HipHop reads under the high confidence (irreproducible discovery rate<0.05; Table S5; see Methods) peaks.”. Therefore, we consider that the aforementioned consensus bias is unlikely the cause.

***Reviewer:*** *•	Given that a main point of the manuscript is to provide a new method for chromatin researchers, the method description is minimal. Mainly, it points to the original Henikoff lab protocol. However, important modifications are not noted, as no antibody incubation should be carried out etc. Given that this is a methods-focused paper, a detailed protocol should be provided either in the manuscript, the supplemental materials, or github.*

**Authors:** A detailed protocol is now included as a Supplemental Method file.

***Reviewer:*** *Experimental details*

*•	The purification of nGFPMNase is insufficiently described. Presumably this construct has a His tag, but this information is not provided, nor is the location of the tag. Was the tag removed from the final protein? Please provide a map for the plasmid, its sequence, and ideally submit the plasmid and sequence to AddGene.*

*•	Bloomington stock numbers change/get removed. Please provide full genotype for the fly lines used.*

*•	Please provide the exact integration site for the HipHop-GFP transgene.*

*•	Details regarding Drosophila culture conditions are missing (humidity, light/dark cycles, etc.).*

**Authors:** The requested information has been added.

***Reviewer:*** *•	The exact timing of the embryo collection is unclear from the description. Were the embryos collected for 8-12hrs and thus 0-12hrs old, or where all embryos between 8 and 12hrs old?*

Authors: clarified as 0-12hrs old embryos.

***Reviewer:*** *•	Regarding the nuclei prep, how was cell debris removed and separated from nuclei?*

**Authors:** We did not implement a separation step. However, since the nuclei were collected by low speed centrifugation, we suspect that some of the cellular debris remained in the supernatant and were eliminated.

***Reviewer:*** *•	In terms of data handling, it appears that peak calling was done independently on each replicate, but then combined. Please clarify how this was done. Also, how were no-tag samples processed and used to “normalize”?*

**Authors:** We did not combine the data for peak calling, we show peaks called for each replicate/treatment separately. The IDR analysis shows the overlap between peaks called between the replicates.

***Reviewer:*** *•	Please make sure that all scripts and analysis pipelines are available on the github page, which is currently not accessible to reviewers.*

**Authors:** updated and available.

***Reviewer:*** *•	Please clarify what is “normalized enrichment” in Figure 2.*

**Authors:** We updated the legends to make it clear that normalized enrichment represents the enrichment (target RPM/Control RPM) of the target protein (HipHop or HOAP) over the no-tag control in RPM.

***Reviewer:*** *•	Table S3 shows up before S2 in the text.*

*•	Typo on line 339 – Tris*

**Authors:** both corrected.

-----------------------------------

Reviewer #2:

***Reviewer:*** *This manuscript describes a very useful variation of the well-established CUT&RUN technique that could simplify the molecular analysis of chromatin protein distribution in complex genomic regions such as the Drosophila telomeric regions. By modifying the classical CU&RUN approach, through the employment of a high affinity nanobody to GFP used to bring the MNase to the binding sites of GFP-tagged chromatin proteins (nanoCUT&RUN), the authors were able to provide a chromatin profiling tool for HOAP and Hiphop telomere capping proteins at Drosophila telomeres. The analysis of the distribution of these telomere capping proteins were indeed consistent with previously performed ChIP-seq analyses, although unlike ChIP, the nanoCUT&RUN is performed in situ avoiding some of the limitations of ChiP, thus confirming the efficiency of the tool.*

*The strategy in the manuscript is well described and the conclusions on the efficiency of the approach are supported by the data. However, although the single analyses of HOAP and Hiphop profiling on telomeric chromatin are informative, I have some concerns regarding to the comparative distribution. This is based on the following considerations:*

*1.	Flies expressing either GFP-HOAP or GFP-Hiphop are of different origin and/or different genetic background (knock-in line for HOAP, transgenic line for GFP-Hiphop in a hiphop mutant background). This should be taken into account when the relative enrichment of these factors is described. This holds true also for the potential centromeric localization of HihHop, as it cannot be excluded that it may depend on the transgenic feature of GFP-HipHop. Without other independent CUT&RUN experiments (with HOAP and Hihhop carrying different tags), authors should be cautious in their interpretations in comparing the distribution profiles in relation to each other.*

**Authors:** We agree with the reviewer that caution is warranted when comparing the distribution patterns of two proteins. We surmise that the differences between using a knock-in allele versus a transgene would likely be at the expression levels of the target protein. However, we cannot exclude the extreme cases where expression driven by a transgene is highly abnormal so that target proteins assume abnormal localizations. Since our GFP-hiphop transgene rescues a null mutation, we suggest that the aforementioned case does not apply here. Nevertheless, we added cautionary notes wherever appropriate to emphasize the need to consider expression levels of different proteins. We add that our method offers a unique opportunity in comparing the profiles from multiple GFP-tagged proteins since our method is based on a common nanobody-GFP interaction, whereas the traditional antibody based methods rely on target specific antibodies. We also add to the revision our recent results showing that hiphop is a recessive suppressor of silencing induced by X centric heterochromatin, which would be consistent with HipHop’s presence at centromeric regions. **On page 15**, it reads: “Interestingly, our recent results showed that a hypomorphic hiphop mutation behaves as a recessive suppressor of heterochromatin-induced Position-Effect-Variation (PEV) [Cui et al. 2021]. Moreover, the specific case of PEV used in that study involves the X centric heterochromatin. These earlier results seem to be consistent with the presence of HipHop in centric heterochromatin as revealed by this study, and with a potential role of HipHop in silencing that is not limited to chromosome ends.”.

***Reviewer:*** *2.	The different genetic background could also explain the different enrichment of HOAP and Hiphop on TART-A that could be underrepresented in the cav knocking line. This should be addressed by sequencing the telomeric DNA from both lines.*

**Authors:** As suggested by the reviewer, we have sequenced the genomes of the two lines as well as the “no-tag” control, and re-analyzed the data. Our major conclusions remain supported by the data.

***Reviewer:*** *Minor concerns*

*1.	On page 11, line 215: please indicate what “three classes” refers to*

*2.	The cloning strategy (plasmids, primer sequences etc..) for the generation of the GFP nanobody should be indicated in the Materials and Methods*

**Authors:** information added.

***Reviewer:*** *3.	When citing previous work on Drosophila telomere capping, I would like to encourage the authors not to be refrained from using the concept of “terminin” which no way detracts from their excellent work done in this field.*

**Authors:** We added references introducing the concepts of “Terminin” in Drosophila and “Sheltarin” in mammals. On page 3, it reads: “Drosophila capping proteins have been collectively called “Terminin” [Raffa et al. 2010], similar to the concept of “Shelterin” proposed for telomerase-maintained systems [de Lange 2005].”.

---------------------------------

Reviewer #3:

***Reviewer:*** *Chen et al. introduce a new elaboration of CUT&RUN that allows facile profiling of genome-wide DNA binding of GFP-tagged chromatin proteins. They demonstrate convincingly the robustness of this method using a well-studied target protein with many known binding sites throughout the Drosophila genome. They next profile two telomere-binding proteins and discover the one such protein localizes not only to chromosome ends but also to the centromere. They also discover unexpected enrichment deep into the subtelomere. Importantly, they map the reads to a highly contiguous genome assembly that includes assembled centromeres, telomeres, and subtelomeres. The method can be applied easily to other GFP-tagged proteins.*

*1.	I urge the authors to establish a stronger rationale for developing the new method. Does CUT&RUN with anti-GFP perform poorly? Does nanoCUT&RUN perform especially well for proteins that bind repetitive genomic regions? What are the drawbacks of current method(s)? The authors mention the advantage of assaying multiple isoforms when isoform-specific antibodies are not available (Results, line 166). I wonder if this aspect of nanoCUT&RUN could be played up in the Introduction AND then demonstrated using the TRL data (see #3 below).*

**Authors:** Our rationale is now discussed more extensively in the Introduction (**on pages 4-5**). The truth of the matter is that many of our chromatin proteins of interest have the GFP tag and we would like to develop a method to streamline the process of profiling their chromatin occupancy. As discussed in the revision, anti-GFP antibodies have its drawback. Based on our collective experiences, a well characterized monoclonal anti-GFP antibody with consistent performance in IP-related experiments has not been widely available. For polyclonal anti-GFP antibodies, one has to deal with batch-to-batch variabilities. **On pages 5-6**, it reads: “In our studies of Drosophila chromatin proteins, we often employed tagging with a Green Fluorescent Protein (GFP) [e.g. Schneiderman et al. 2012; Wesolowska et al. 2013; Tang et al. 2017; Huang et al. 2021] so that live imaging of the target proteins could be achieved as long as the GFP-tagged target had been proven functional in genetic rescuing experiments. In theory, an anti-GFP antibody could be used to conduct Cut&Run profiling of any such target. However, our collective experiences with monoclonal anti-GFP antibodies have been unsatisfactory. Since single-domain anti-GFP nanobodies offer consistent performance in IP related experiments [e.g. Cicconi et al. 2017; Donohoe et al. 2018], and can be readily purified from bacteria, we were prompted to develop a method generally applicable to profiling GFP-tagged chromatin proteins .”

***Reviewer:*** *2.	In the Introduction, the authors state “how capping proteins are recruited to telomeres remains obscure.” The authors also assert that the sequence-independent localization of these proteins has been appreciated for 30 years. However, the data presented did not offer much new insight into the “how.” The data are certainly suggestive of a potential for HipHop to bind nucleosomes and but additional support is required to draw a stronger inference (see comment 7). In the absence of additional supporting data, I suggest that the authors reconsider posing this question early on. Instead, the authors could highlight how the new method is especially useful for addressing a specific question about telomeres.*

*I wonder if the Introduction would be much stronger if the authors follow the flow of*

*the abstract, which leads with the technology. I suggest making the telomere biology secondary in the Introduction (as was done in the abstract).*

**Authors:** The goal of profiling telomere proteins was to help answer the question of “how”. Available evidence suggests that capping protein binding to telomeres is sequence-independent in Drosophila. However, as we suggested in the introduction that natural binding partners of the capping proteins are these retrotransposons. Therefore, one cannot rule out the hypothesis that there are “preferred” binding sites on the transposons that the capping proteins rely on for proper localization, perhaps to initiate binding. Even though our data add more evidence against this hypothesis, we argue that knowing the profilings on natural chromosomes could have shed light on the question of “how”.

***Reviewer:*** *3.	Given that the authors mapped the HOAP and HipHop-associated reads to the heterochromatin-enriched assembly to look at binding to repetitive genomic regions, I wondered why the authors chose not to map the TRL-long isoform reads to the heterochromatin-rich assembly. I appreciate that TRL is used primarily to validate the method, and previous data did not sample these regions of the genome, but it does seem like a missed opportunity. Moreover, the contrast of TRL-short vs TRL-long binding profiles would allow the authors to demonstrate in a compelling way the power of nanoCUT&RUN when isoform-specific antibodies are not available. And even if isoform-specific antibodies are available, it seems to me that probing for common tag with the same antibody (i.e., GFP) offers a more controlled experiment than probing multiple isoforms with different antibodies. Might adding this analysis and highlighting its power in the introduction offer a key motivation to develop nanoCUT&RUN?*

**Authors:** We appreciate the desire of the reviewer to take the fullest advantage of the TRL data. However, we felt strongly that our analyses on TRL, although sufficient for a proof of principle experiment, might not be sufficient for probing the biology of TRL and heterochromatin. Clearly additional experimentation would be needed for validation, had we expanded those analyses and drawn conclusions based on them. Since our resources are limited, we believe that what the reviewer suggested would be more suitable for another study focusing on TRL.

***Reviewer:*** *4.	There were several references to divergence between the PacBio genome assembly line and the experimental line (for example, an absence of reads mapping to parts of the retrotransposon consensus sequences). How else might divergence between the experimental strain and genome assembly affect the inferences? I’m thinking particularly about the heterogeneity of peaks observed along different chromosomes in Figure 2 and Figure S3. Could the reads from the input be used to address divergence from the consensus sequences?*

**Authors:** We have sequenced genomic DNA from the experimental strains to gain a better understanding of the sequence divergence. These new datasets identified where there is likely divergence in repeat identity and copy number between strains and we now show in new supplemental figures S5 and S9 how the genomic sequence diverges. These results help to distinguish between sequence variation and difference in telomere protein enrichment. Our conclusions remain unchanged about the broad distribution of telomere proteins. We add a discussion of these results **on page 12**. It reads: “Because the fly strains may differ in their organization and sequence of telomeric regions, and we map to a reference genome assembly, we sequenced a genomic DNA control for each fly strain expressing GFP-HipHop or GFP-HOAP, and the no-tag control. The read distribution is shown in Figure S5. We do see some variation in genomic background between the strains such that differences between HOAP and HipHop on telomere 3L are driven by structural variation (Figure S5B). However, the overall pattern of HipHop and HOAP enrichment at the telomeres is the same.”.

***Reviewer:*** *5.	In Line 233, it is stated that “the enrichment extends beyond the HTT domain and into the subtelomeric region >100kb from the distal…”. However, in Line 248, the authors assert that the binding pattern from the nanoCUT&RUN is similar to previous data showing only localization across the terminal 11kb of the chromosome. Please clarify.*

**Authors:** We deleted the comparison with our prior ChIP results using a white-marked telomere.

***Reviewer:*** *6.	I am not a specialist in chromatin protein—nucleosome interactions, but I suspect that additional data is required to support the interesting claim that HipHop may interact with nucleosomes. Can the authors map nucleosomes in the same strain to address the possibility that HipHop binding corresponds to nucleosomes? Related: Is this base pair- resolution another benefit to using to nanoCUT&RUN? If so, might it be worth anticipating this result/advantage in the Introduction about the drawbacks of current methods.*

**Authors:** We agree with the reviewer that additional data are needed to support our claim, and deleted our proposition that HipHop might interact with the nucleosomes.

***Reviewer:*** *7.	I encourage the authors to make their telomere figures easier to read (Figure 2, S3). Consider contracting the different non-telomeric transposon categories so that there are fewer colors. Also, the tracks at the bottom could be much larger. I also suggest designating clearly which side of the plot corresponds to the telomere and which side is facing the centromere. Finally, using black and grey to refer to single vs multi-mapping reads is confusing given that these colors correspond to specific sequence features as well (simple satellites and other). Similarly, Figure 4 uses orange for the peak calling and “Other Jockey.”*

**Authors:** We thank the reviewer for their suggestions. We changed most colors so that they do not repeat. We are reluctant to add additional colors, so we elected to keep the black and grey because they are in different parts of the figure, which we hope reduces confusion. We now annotate which side of the contig is the telomere end.

***Reviewer:*** *8.	Is it surprising that the multi-mapping reads show a lower pile up than the unique reads in Figure2? What are the implications of this?*

**Authors:** This is not surprising, as those grey regions largely correspond to tandem repeats with low mappability. RPM is calculated separately for all reads and uniquely mapped reads. Because the total number of mapped reads will be larger for ‘all reads’ than ‘uniquely mapped reads’, RPM should be higher for uniquely mapped reads in general.

***Reviewer:*** *9.	Figure S2D suggests that HOAP is at the centromere too. If so, why was this observation not highlighted in section 4? Also, what makes up the “other” category in Figure S2B and D?*

**Authors:** “Other” is any place in the genome that is not centromere or telomere, and it looks to be primarily driven by the Y chromosome signal. As stated in revised legend: in this category most of the peaks actually localized on one Y-linked scaffold (Y_scaffold4), which is also enriched in HTT, however this scaffold is unlikely to represent the Y telomere because of its cytological location. The y-axis in fig S2B and D is total count (not normalized for size of the region), and HipHop has many more. We updated the y-axis label to be clear.

***Reviewer:*** *Minor comments:*

*•	Why does the localization to HeT-A sublineages suggest a lack of sequence-specificity (lines 225-227) rather than the data showing localization to three very different major retrotransposon lineages?*

**Authors:** There’s no obvious region that jumps out as being a special binding site for these proteins. We aren’t really sure what the reviewer’s point is about the three very different major retrotransposon lineages? We modified the sentence to read: “This suggests that HipHop might not have a strongly preferred DNA sequence **within each HTT element** for binding”.

***Reviewer:*** *•	Line 115: “…calcium as the original MNase…” seems like an awkward construction. Consider revising.*

**Authors:** revised.

-----------------------
