# Supplementary material for: The nanoCUT&RUN technique visualizes telomeric chromatin in Drosophila
Source: PLoS Genet. 2022 Sep 1;18(9):e1010351. doi: 10.1371/journal.pgen.1010351 (PMC9473618; doi:10.1371/journal.pgen.1010351)
Supplement: Attachment — Submitted filename: ChenRebuttal2.2.docx [file pgen.1010351.s022.docx]

Authors’ responses to reviewers’ comments

Reviewers’ comments have italicized, and changes made to the text has been highlighted in red.

**Reviewer #1**

***Reviewer****: Line 61: Consider adding, “unlike the sequence-specific binding of Shelterin components in mammals, Drosophila Terminin proteins are sequence-independent."*

**Authors**: sentence added.

***Reviewer****: Line 112: Consider defining “nanobody”*

**Authors**: a definition added.

***Reviewer****: Line 162: How many peaks were previously called? Stating “All of these profiles show peaks” makes it unclear where there are three peaks or 3,000 peaks that are recapitulated by the TRL-S and TRL-L profiling.*

**Authors**: The number of peaks called previously and the extent of overlapping with our data are presented in a new Supplemental Figure S2.

***Reviewer****: Line 234: map -> mapped*

**Authors**: Corrected.

***Reviewer****: Line 271: While expression certainly could explain the heterogeneity, could elevated read number for HOAP at TART-B and Het-A5 could also be related to sequence specificity?*

**Authors**: It is possible that the pattern is driven by preferential binding of HOAP to TART-B and Het-A5, however as we don’t have evidence for sequence specificity, it’s not clear how that is true. It could however be driven by differences in HOAP enrichment among locations. For example, HOAP could be more enriched in a region that contains more TART-B and Het-A5. It is difficult to distinguish between these possibilities. We edited this section to say: “The exception for TART-B and Het-A5 may be driven by variation in HOAP enrichment within or between telomeres, although we cannot exclude the possibility that HOAP has a sequence preference for these elements.”

***Reviewer****: I am still unable to read the legend of Figure 4 – please make the boxes larger.*

**Authors**: We edited the legend to be larger and the same size for Figures 2 and 4. We hope that they are now easy to read. Thanks for letting us know.

--------------------------

**Reviewer #2**

***Reviewer****: The authors use TRL profiling as proof-of-principle to validate nanoCUT&RUN as a technique for profiling DNA-binding proteins. Therefore, a direct comparison of nanoCUT&RUN profiles with published TRL ChIP-seq or CUT&RUN datasets is needed. The authors show individual TRL binding events in Fig 1b; however, genome-wide comparisons are required.*

**Authors:** We have added a Supplementary Figure S2 where we compare nanoCUT&RUN profiling to ChIP-seq profiling of TRL. We added a comment in the text to read: “About 48% of TRL-short and 57% of TRL-long peaks overlap with sites identified by ChIP-seq [Gaskill et al. 2021] (Figure S2), confirming that nanoCUT&RUN detects bone fide TRL binding sites.”.

***Reviewer****: The utility of nanoCUT&RUN to the scientific community will be maximized by sharing the materials and protocols required for generating nGFPMNase.*

*o Currently, the authors include a graphical map of the vector used to produce recombinant protein. It is necessary that the sequence of this vector be published as part of the manuscript, rather than being made available upon request. Ideally, the vector itself would be deposited where it can be acquired by the community, such as the Addgene vector database.*

*o Currently, the protocol used for inducing and purifying nGFPMNase is not described in any detail. It is necessary that all possible detail be included so that others can replicate their approach. The technical advance is a major part of this manuscript’s significance; other labs should be able to employ nanoCUT&RUN after reading this paper.*

**Authors**: The vector along with its sequence has been deposited to Addgene (ID: 187826). A new Supplemental file has been added to present a detailed purification protocol.

***Reviewer****: High-throughput sequencing approaches to studying telomere biology are hampered by highly repetitive DNA sequences. Although the new HipHop and HOAP findings are primarily descriptive in nature, they suggest that HipHop and HOAP do not bind telomeres in a sequence specific manner because the authors find no evidence of a motif and the DNA binding profiles are broad rather than peak-like (as is typically observed for transcription factors). The authors should plot their own nanoCUT&RUN TRL data at telomeric sequences; this could potentially provide a contrasting DNA binding pattern (ie. Peak-like, or absent), which would considerably strengthen the conclusion that HipHop and HOAP bind broadly across telomeres and mitigate concerns that their DNA binding pattern is due to technical rather than biological reasons.*

**Authors**: We acknowledge that it would be ideal to contrast narrow transcription factor peaks with the broad HipHop and HOAP peaks at telomeres. However, TRL does not localize to the telomeres, so it is not feasible to compare the profiles of TRL to HipHop and HOAP. Unfortunately, we do not have data for a transcription factor that binds in the same region as HipHop and HOAP.

-------------------------
